# Substance-Related Acute Toxicity Deaths in Canada From 2016 to 2017: Protocol for a Retrospective Chart Review Study of Coroner and Medical Examiner Files

**DOI:** 10.2196/49981

**Published:** 2025-04-10

**Authors:** Jenny Rotondo, Amanda VanSteelandt, Fiona Kouyoumdjian, Matthew J Bowes, Tanya Kakkar, Graham Jones, Brandi Abele, Regan Murray, Emily Schleihauf, Jessica Halverson, Jennifer Leason, Dirk Huyer, Beth Jackson, Songul Bozat-Emre, Devanshi Shah, Erin E Rees

**Affiliations:** 1 Substance-Related Harms Division Health Promotion and Chronic Disease Prevention Branch Public Health Agency of Canada Ottawa, ON Canada; 2 Ontario Ministry of Health Toronto, ON Canada; 3 Nova Scotia Medical Examiner Service Department of Justice Dartmouth, NS Canada; 4 Department of Laboratory Medicine and Pathology Faculty of Medicine and Dentistry University of Alberta Edmonton, AB Canada; 5 Board of Directors Canadian Association of People Who Use Drugs Saskatoon, SK Canada; 6 Collaboration on Death Investigation Data Health Promotion and Chronic Disease Prevention Branch Public Health Agency of Canada Ottawa, ON Canada; 7 Canadian Public Health Service Emergency Management Branch Public Health Agency of Canada Ottawa, ON Canada; 8 Migration Health Branch Immigration, Refugees and Citizenship Canada Ottawa, ON Canada; 9 Department of Anthropology and Archaeology University of Calgary Calgary, AB Canada; 10 Office of the Chief Coroner for Ontario Ontario Forensic Pathology Service Toronto, ON Canada; 11 Health Equity Policy Division Strategic Policy Branch Public Health Agency of Canada Ottawa, ON Canada; 12 Provincial Information Management and Analytics Manitoba Health Winnipeg, MB Canada; 13 Community Health Sciences Max Rady College of Medicine, Rady Faculty of Health Sciences University of Manitoba Winnipeg, MB Canada; 14 Public Health Risk Sciences Division National Microbiology Laboratory Public Health Agency of Canada Saint-Hyacinthe, QC Canada

**Keywords:** acute toxicity, Canada, chart review study, coroner, death investigations, drug overdose, medical examiner, mortality, poisoning, protocol

## Abstract

**Background:**

Canada continues to experience a national overdose crisis. While studies are available at the regional, provincial, and territorial levels, detailed national data regarding the burden and context of substance-related acute toxicity deaths are limited, particularly in subpopulations. In response to the overdose crisis, the Public Health Agency of Canada, in collaboration with provincial and territorial ministries of health and chief coroner and chief medical examiner offices, has undertaken a national chart review study.

**Objective:**

This study was conducted to describe and compare the characteristics of substance-related acute toxicity deaths that occurred in Canada between 2016 and 2017, including descriptions of those who died, the substances involved, and the circumstances surrounding their deaths. This paper describes the study methodology in detail.

**Methods:**

This retrospective, population-based, and cross-sectional study involved the review of coroner and medical examiner files for deaths that met the study case definition. Data were collected on demographic and socioeconomic characteristics, medical and substance use history, proximal circumstances surrounding the death, and toxicology findings using a standardized data collection tool that underwent 2 pilot studies. Data abstractors underwent training and regular intrarater reliability exercises with a fictitious death investigation file. Data quality was assessed based on the consistency of abstractor intrarater reliability scores and the completeness of core variables and variables for key concepts. Data were linked to national datasets to allow for the examination of area-level geographic and socioeconomic characteristics. Descriptive analyses will examine differences across subpopulations and the general Canadian population. Latent class, spatiotemporal, qualitative, and premature death analyses are also planned. Where possible, analyses will be stratified by the manner of death and sex.

**Results:**

The study began in the summer of 2018, and abstraction was delayed due to the COVID-19 pandemic. All activities are expected to be completed by early 2025. A total of 9414 coroner and medical examiner files met the study case definition. Most abstractors (25/26, 96%) met the established threshold for consistency throughout abstraction without the need for remedial training. In general, core study variables, including geographic variables and substances contributing to death, had very good availability. Study variables related to the person’s health, history of substance use, and events surrounding the acute toxicity event were available for most records. Socioeconomic variables and variables describing socially constructed identities and potentially traumatic life events were mostly unavailable.

**Conclusions:**

This study provides the most detailed national information on substance-related acute toxicity deaths in Canada to date and can serve as a pre–COVID-19 pandemic baseline for assessing the evolution of the overdose crisis. Results can inform policies and programs to address the overdose crisis, the development of common approaches to medicolegal death investigations, and future research activities.

## Introduction

### Background

Canada continues to experience a national overdose crisis, with a large burden of harms that are inequitably distributed throughout the population. Between January 2016 and June 2023, a total of 40,642 people died of opioid toxicity alone, with associated mortality rates increasing over time [1]. Compared to other countries where data are available, the United States and Canada have seen rapid escalations and persistently high accidental acute toxicity mortality rates [2]. Information on the characteristics and circumstances of those who have died is critical to informing and evaluating strategies aimed at preventing harms.

High-quality data are particularly needed at the national level to estimate burden and explore acute toxicity deaths among subpopulations, thereby enhancing our understanding of inequities and risk factors [3-6]. Although numerous studies and reports have described substance-related acute toxicity deaths at the regional, provincial, and territorial levels [7-24], they used a variety of methods and case definitions, making it difficult to aggregate findings at the national level and compare them across provinces and territories. Vital statistics data from medical certificates of death are standardized and routinely collated at the national level in Canada [25]. While this is a common source for acute toxicity mortality data [26,27], it lacks detailed information on the characteristics of those who died and the specific substances that contributed to their deaths [6,28,29].

By contrast, coroners and medical examiners (CMEs) collect demographic, socioeconomic, health, and other risk factor data during death investigations, as well as information on the circumstances of the death and toxicological findings. Although national opioid toxicity mortality surveillance uses CME data reported by provincial and territorial ministries of health to the Public Health Agency of Canada (PHAC), data are submitted at an aggregate level by some ministries, with stratification by a small number of variables of interest [1]. As demonstrated in similar studies, additional valuable information can be gleaned from complete CME files [30-32].

### Objectives

Our study objective was to describe and compare the characteristics of substance-related acute toxicity deaths that occurred in Canada in 2016 and 2017, including descriptions of those who died, the substances involved, and the circumstances surrounding their death. This paper describes the study methodology in detail, as well as the process for developing study material and findings regarding variable completion. It may be used as a reference document and to support the development of similar studies in the future.

## Methods

### Study Design

This retrospective, population-based, and cross-sectional study involved a review of the CME files of people who died of substance-related acute toxicity in Canada between 2016 and 2017. Upon identifying CME files that met the study case definition, data were abstracted using a standardized data collection tool.

### Personnel

At the study outset, the PHAC study team assembled a coinvestigator team to inform the development and rollout of the study protocol, analysis planning, interpretation of findings, and production of knowledge translation products. The coinvestigator team consisted of 14 people with expertise in the CME system, death investigations, toxicology, pharmacology, social determinants of health, surveillance, epidemiology, harm reduction, the health of Indigenous people, qualitative research, corrections, social services, medicine, provincial and territorial health authorities, the federal Health Portfolio, public health in Canada, and lived experience with substance use. Coinvestigators were also well connected with other substance-related surveillance, research, and prevention activities occurring in Canada.

In addition to the PHAC study and coinvestigator teams, data abstractors and students were recruited to support data collection, preparation, and analysis activities. Additional subject matter experts were invited to participate in analysis-specific project teams. For example, people with lived experience and people with expertise in youth and older adult health, suicide, emergency medicine, substance use, pain research, race and ethnicity studies, addiction medicine, and corrections have joined specific projects focused on these topics. Finally, a team of Indigenous researchers worked with coinvestigators and project team members to develop products focused on First Nations, Métis, and Inuit people who died due to acute toxicity, with a focus on protective factors.

### Literature Review

To inform the development of the study protocol, the data collection tool, and planned analyses, a literature review was conducted to identify knowledge gaps with respect to substance-related acute toxicity deaths at the national level in Canada. Collaborating with a librarian, a search strategy with inclusion and exclusion criteria and filters was developed. The MEDLINE, Embase, and SCOPUS databases were searched, and 2 team members reviewed titles and abstracts for relevance. A gray literature search was also conducted to identify relevant reports not published in peer-reviewed journals. Finally, additional articles were identified by reviewing the reference lists of relevant articles and reports. Information was abstracted using a standardized data collection tool and synthesized based on themes.

Although the review concentrated on the Canadian context, evidence from other countries was drawn on when limited Canadian data were available. The review focused primarily on substance use; pharmaceutical prescribing; risk factors; proximal circumstances; and severe harms related to substance use, including emergency department visits, hospitalizations, and deaths.

### Consultations

In addition to the literature review and expertise of the coinvestigator team, the study’s design and rollout were informed by consultations with various groups, including chief coroners and chief medical examiners (CCCMEs), CCCME office staff, PHAC public health officers, other government of Canada departments (including Health Canada and Indigenous Services Canada), and staff within provincial and territorial ministries of health. The PHAC study team also consulted with national Indigenous organizations during the planning and analysis phases of the study, as well as a Government of Canada council of people with lived and living experience during the analysis phase.

### Study Population

The target population for this study included all people who died in Canada in 2016 and 2017 due to acute toxicity. The accessible population included people whose deaths (1) were investigated by a CME, (2) had a CME report available for data abstraction, and (3) were identified as meeting the case definition. Given the resource intensity of conducting a nationwide chart review study, only 2 years’ worth of data were collected; 2016 and 2017 were selected, as these were the latest 2 years for which data were likely available when work on the study began in 2018.

### Case Definition

The study case definition was any individual who died in Canada between January 1, 2016, and December 31, 2017, after an acute intoxication or toxicity resulting from the direct effects of the administration of exogenous substances where ≥1 of the substances was a drug or alcohol. The study case definition was based on the national CME case definition for an apparent opioid-related death (as of March 1, 2017) [1]. “Acute” refers to adverse health effects that occur within a short period (measured in minutes, hours, or days) following dosage [33]. Substances included alcohol; controlled, illegal, and prescription drugs; new psychoactive substances [34]; over-the-counter pharmaceutical products; and chemicals not intended for human use (eg, nonpharmaceutical inhalants, industrial or household chemicals, or veterinary drugs). Inclusion and exclusion criteria are described in Textbox 1.

**Inclusion and exclusion criteria for our national chart review study of substance-related acute toxicity deaths.**

**Inclusion criteria**

- The death occurred in the reporting province or territory.
- The death occurred in 2016 or 2017.
- The investigation is either ongoing (open, preliminary, or active) or closed (certified or completed).
- The manner of death was deemed an accident (unintentional), suicide (intentional), or undetermined.
- According to the (1) death certificate, (2) autopsy summary, or (3) coroner or medical examiner report, acute toxicity resulting from the direct effects of the administration of exogenous substances where ≥1 of the substances was a drug or alcohol was identified as having caused or contributed to the death.

**Exclusion criteria**

- The death was solely caused by any of the following: chronic substance use (while people who died only due to the effects of chronic substance use were excluded from this study, people who experienced chronic substance use but died due to acute toxicity were included), medical assistance in dying, palliative or comfort care, homicide, occupational exposure, trauma where an intoxicant contributed to the circumstances of the injury (eg, death caused by a motor vehicle collision involving a driver impaired by an intoxicant), other adverse drug effects (eg, anaphylactic shock), or acute toxicity due to products of combustion (eg, carbon monoxide, carbon dioxide, sulfur dioxide, and nitrogen oxides).

### Data Sources

Legislation exists in all Canadian provinces and territories requiring that deaths believed to be the result of violence, accident, suicide, negligence, misconduct, or malpractice be reported to a CME. These acts also require that a CME investigate these deaths to establish the identity of the deceased; the date, time, and place of death; the circumstances under which the death occurred; the cause of death; and the manner of death. Case files generally include the CME’s medical certificate of death, the CME’s summary report, toxicology test results, an autopsy or external examination report (if performed), and continuation notes (file communication records). In addition, they may include a police report, an emergency medical service record, the decedent’s recent medical history (the period differs across CMEs, provinces, and territories), scene notes and photographs, and correspondence with individuals relevant to the case (eg, family physicians or other professionals who knew the person who died). Although there are similarities in how CME investigations are conducted across Canada, the information collected varies over time across provinces and territories, the investigating CME, and the circumstances of the death [35].

The primary source of data for this study was physical and electronic CME files. While every effort was made to obtain complete information for each case, physical files were not always accessible, and the COVID-19 pandemic resulted in additional challenges to the review of case files. For all British Columbia cases and some Ontario cases, data were only obtained from electronic coroner databases and mapped to corresponding variables in the study data collection tool where variables were equivalent. Additional population size and area-level data were obtained from Statistics Canada’s 2016 Census [36], Statistics Canada’s Postal Code Conversion File Plus [37], Statistics Canada’s Index of Remoteness [38], Statistics Canada’s Canadian Index of Multiple Deprivation [39], and Environment and Climate Change Canada [40] to support specific analyses.

### Data Collection Tool

Potential variables of interest were identified from (1) the study’s literature review, (2) CME investigative tools already in use by provinces and territories, and (3) consultations with key stakeholders. Potential variables’ usefulness, relevance to research questions, comparability with other studies, and privacy concerns were considered before their inclusion in the data collection tool. As one of the study goals was to report on data availability within CME files, variables of interest with low expected availability were not excluded. Data were collected on demographic, socioeconomic, and risk factors; drug, medical, and substance use history; proximal circumstances surrounding the death; and toxicology findings for each person who died (Multimedia Appendix 1 provides the variable list). Identifying variables, such as the name and address of the person who died, were collected only if requested by the CCCME office for internal use and were not sent to the PHAC study team.

A standardized data collection tool was built using Microsoft Access (Multimedia Appendix 2 provides screenshots of the tool), and an accompanying detailed user manual and data dictionary were created to enhance data quality and consistency. The data collection tool and accompanying material were pilot-tested twice in 6 provinces and further refined before the official start of the study.

### Data Collection

To facilitate data access and sharing between CCCME office staff and the PHAC study team, 13 data-sharing or research agreements were developed between the PHAC and each CCCME office. Data sharing agreements were also developed between the PHAC and several provincial and territorial ministries of health if required.

Data collection was conducted by individuals approved by collaborators within each CCCME office, including public health officers, CCCME office staff, nurses, epidemiologists, health information analysts, and health sciences and epidemiology students. All abstractors completed a privacy course; received extensive standardized training on how to collect data from CME investigation files; and were given time to review the data collection tool, user manual, and data dictionary. Abstractors were also required to demonstrate their ability to abstract data using a fictitious test case file before data collection began.

Case identification was done using available information systems. Initial broad searches were conducted by CCCME offices or data abstractors to identify anyone who could possibly meet the study case definition. Data abstractors then determined whether each case met the case definition while reviewing the CME files. In offices where it was possible to identify potential cases based on an *International Classification of Disease, 10th Revision*, underlying cause of death code, relevant categories included X40 to X45, X47, and X49 (accidental poisoning); X60 to X65, X67, and X69 (intentional self-poisoning); and Y10-Y15, Y17, and Y19 (poisoning with undetermined intent) [41].

As part of the data collection process, abstractors were instructed to regularly run built-in queries to check data completion. This supported quality assurance activities and decreased the need to return to CCCME offices for further data collection.

### Ethical Considerations

Study data management activities were developed to ensure data confidentiality. All data collected for the study existed electronically in password-protected datasets that were transferred to the PHAC study team via a secure data transfer method. Received data were stored in a restricted PHAC network folder accessible only to study staff responsible for data management or analysis. All study coinvestigators and data abstractors were asked to sign confidentiality agreements.

To ensure confidentiality in knowledge translation products, random rounding to base 3 has been applied to the raw counts of all tables displaying results, counts <10 will be suppressed, and variable categories will be collapsed where appropriate. In random rounding to base 3, values that are multiples of 3 do not change, and those that are not multiples of 3 have a two-thirds chance of rounding to the nearest multiple of 3 and a one-third chance of rounding to the second nearest multiple of 3 [42]. This will perturb the count to within 2 units of the true value. As individual counts, subtotals, and totals are independently rounded, they may not add up when summed. Percentages, which are calculated using the rounded values, also may not add up to 100%. All study products will be audited to ensure that the same cell value is rounded in the same direction across tables.

As the study was part of a time-limited, exploratory research activity, research ethics board review and approval were sought. The study protocol was reviewed and approved by the Health Canada and PHAC Research Ethics Board (REB 2018-027P), the University of Manitoba Health Research Ethics Board (HS22710), and the Newfoundland and Labrador Health Research Ethics Board (20200153). It also underwent privacy assessments by the PHAC; the Manitoba Health, Seniors, and Active Living Health Information Privacy Committee; and the British Columbia Office of the Chief Information Officer.

### Data Preparation

Collected data underwent multiple stages of preparation, including case confirmation, comment review, redundancy checks, and variable cleaning. In situations where abstractors were unsure whether a case met the study case definition, they were asked to provide a nonidentifying description of the situation, which was shared with a subset of the coinvestigator team for decision-making. Similarly, when abstractors were unsure of how to record information from a case file, they were instructed to leave detailed comments in the database explaining the issue. These comments were later reviewed by the PHAC study team, and corresponding variables were adjusted as required.

Because the data collection tool had to allow abstractors to abstract data in a variety of formats, the database contained redundant variables that captured the same concept in slightly different ways. The study team reviewed the logic of redundant variables to ensure that variables with missing information were completed with information from redundant variables where possible. For example, if a person was documented to have been enrolled in an opioid agonist treatment program, their medical history was updated to include a history of substance use disorder.

Finally, the dataset underwent extensive data cleaning activities, particularly for values reported in free-text fields. Free-text fields were reviewed by the study team to correct spelling and match with existing variable values missed by abstractors. For more complex variables, the entered values were categorized according to a framework to simplify future analyses (eg, specific medical conditions were categorized according to types of medical conditions relevant to acute toxicity). Finally, some free-text variables were left “as is” for future thematic analysis.

### Data Quality Indicators

#### Consistency of Data Collection

To ensure that data were collected in a consistent manner across data abstractors and throughout the data collection period, abstractors were required to complete an intrarater reliability (IaRR) exercise. After completing training activities and before beginning data collection, each abstractor was asked to abstract data from a fictitious death investigation file, and the abstracted data were compared to a gold standard. This was done again at the midpoint and at the end of data collection, allowing for the IaRR to be assessed at these points. Additional IaRR assessments were also conducted between any breaks in data collection, if required.

To develop the gold standard, study coinvestigators and PHAC study team members created a fictitious CME test file. Next, several PHAC study team members not involved in data collection independently used the data collection tool to collect data from the CME test file. The data collected were reviewed by members of the coinvestigator and PHAC study teams to identify inconsistencies in what was collected and to come to a consensus regarding what should have been captured in the data collection tool’s fields. Because the data collection tool includes hundreds of fields, it was not optimal to base IaRR measurements on all data variables. Instead, the IaRR measurements were based on a subset of variables that were deemed to be of critical importance, as well as variables for which data collection was inconsistent during the pilot phase.

As variables were mostly categorical, IaRR was measured using Cohen κ, which accounts for the extent of agreement possible, having controlled for consensus by chance [43]. In instances where variables were not categorical (eg, dates), κ was based on whether the abstracted fields were consistent with the gold standard. The abstracted field was considered consistent only if blank variables in the gold standard were also blank in the data abstracted during the IaRR exercise or if the IaRR exercise data captured the same information in the same variable field as the gold standard. All other scenarios would deem the field to be discordant with the gold standard. This approach dichotomized nonnominal data so that they could contribute to κ coefficient calculations.

The baseline measurement for each data abstractor was taken before starting data collection for the study. No specific feedback on their performance was provided, as they were asked to abstract the same file again over the course of data collection for the IaRR assessments. Generally, a minimum value of 0.60 was expected for κ values calculated against the gold standard, as per the approach recommended by Landis and Koch [44]; values below this threshold resulted in remedial abstraction training (Table 1). IaRR κ values calculated against previous time point measurements were considered alongside the IaRR κ values calculated against the gold standard to determine the need for remedial training at each time point. This was to ensure that any decreases to the κ value were appropriately interpreted in cases where discordances between time points resulted from incorrect responses being changed to correct responses.

**Table 1:** How data abstractor intrarater reliability κ values were interpreted for our national chart review study of substance-related acute toxicity deaths.

κ			Actions
Test vs gold standard	Test between time points		
Low (<0.60)	High (≥0.60)	Abstractor is consistent across time points, but accuracy is problematic. Remedial action is required.	
High (≥0.60)	Low (<0.60)	Abstractor is exhibiting improved accuracy. Provided that the gold standard κ is >0.60, no remedial action is required.	
Low (<0.60)	Low (<0.60)	Abstractor is not exhibiting acceptable accuracy. Further investigation is needed to determine why IaRR κ remains low and why remedial action is required.	
High (≥0.60)	High (≥0.60)	Abstractor exhibits consistent, high-quality abstraction. No remedial action is required.	

#### Data Completeness

The availability of information for core variables and a set of variables capturing key concepts in the data collection tool was used as an indicator of data completeness. For each data source, this was measured as the proportion of records missing information for each core variable and key concept.

### Data Analysis

The variables selected for analysis were based on either 1) hypothesized relationships with substance use, acute toxicity, and acute toxicity mortality, or 2) as theoretical intervention points to prevent acute toxicity deaths (eg, contacts with the health care system). Descriptive statistics were used to compare subgroups among people who died of acute toxicity (within the dataset), as well as to compare people who died of acute toxicity with the general Canadian population. Data obtained through linkage to Statistics Canada’s Postal Code Conversion File Plus [37] and the Canadian Index of Multiple Deprivation [39] based on the postal code or municipality of residence, acute toxicity event, and death allows for the assessment of area-level geographic and socioeconomic characteristics.

Many of the study’s variables have missing data because only information that was available in CME files was used for data abstraction. As cases with missing data will not be removed from analyses unless they are systematically unavailable for a province or territory, most of the descriptive analyses will present minimum proportions or rates; that is, the minimum number of people who died of acute toxicity with a given characteristic. This is an important consideration for comparisons with the general population, as proportions or rates from the study population that are lower than those observed in the general population may be due to missing data. The true value could be lower than, equal to, or higher than the national statistic. However, if the study population’s proportion or rate is higher than that of the general population, we expect that the true value is at least as high as that observed in the study. In situations where it is known that a variable was only available for select provinces and territories, subnational analyses may be performed.

More complex analyses of the study dataset are also planned. These include using latent class analysis to identify subpopulations among those who died of acute toxicity, spatiotemporal analysis to determine whether geographic and temporal clustering exists in the distribution of acute toxicity deaths, and a case-crossover analysis of weather patterns and acute toxicity deaths. Several free-text variables will also be used for thematic qualitative analyses. Finally, data from the study will be compared with other sources of Canadian mortality data (national surveillance data on apparent opioid-related deaths and Vital Statistics data) [1,25] to measure case ascertainment and premature mortality.

Where possible, analyses of the study dataset have been stratified by the manner of death and used the “sex- and gender-based analysis plus” approach to understand how intersectional identities, histories, and the distribution of resources contribute to acute toxicity deaths [45].

## Results

### Study Timeline

Work on the study began in the summer of 2018, and all activities are expected to be completed in 2025 (Textbox 2). The extended lag between the start of the study and the dissemination of findings is related to several factors. This ambitious project was the first research collaboration between the PHAC and all CCCME offices across the country. As these were new partnerships, time was invested in developing data-sharing agreements and seeking approvals from research ethics boards, privacy committees, and provincial and territorial officials. The nature of the study itself, a chart review, required significant personnel and time resources to review files, abstract, and prepare data, with each case file taking approximately 60 (range 30-100) minutes to review. Finally, study progress was also impacted by the COVID-19 pandemic, which diverted resources and significantly interrupted data collection, particularly where abstractors were working with physical files in office locations.

**Study timeline for our national chart review study of substance-related acute toxicity deaths.**

- August 2018 to October 2019: Consultations, planning, data tool and documentation development and piloting, and research approvals (including departmental, scientific, security, research ethics board, and privacy reviews)
- February 2019 to March 2022: Development of data-sharing agreements with chief coroner and chief medical examiner offices
- March 2019 to October 2022: Abstractor training and data collection
- October 2019 to November 2022: Data preparation
- May 2022 to December 2024: Data analysis and knowledge translation
- June 2022 to September 2022: Release of preliminary results [46,47]
- December 2022: National summary report [48]
- January 2023 to December 2025: Peer-reviewed publications [49-52]
- May 2023 to March 2025: Evaluation

### Available Files

A total of 9414 CME files met the case definition and were accessible for the study (Table 2). The records available from British Columbia included only deaths due to “street drugs” or diverted prescriptions that were accidental or had an undetermined manner of death. As such, data for people who experienced acute toxicity deaths by suicide or due solely to prescribed substances or alcohol were not available. On the basis of a report released by the British Columbia Coroners Service, there were approximately 199 deaths due to suicide by poisoning in 2016 and 2017 that may have met this study’s case definition [53].

**Table 2:** Study population size^a^ and data sources by jurisdiction and year for our national chart review study of substance-related acute toxicity deaths.

Province or territory	2016 deaths (n=4164), n (%)	2017 deaths (n=5247), n (%)	Total deaths (N=9414), n (%)	Data sources
British Columbia^b^	993 (23.85)	1494 (28.47)	2487 (26.42)	Electronic database
Alberta	807 (19.38)	951 (18.12)	1758 (18.67)	Physical files
Saskatchewan	123 (2.95)	123 (2.34)	246 (2.61)	Physical and electronic files
Manitoba	180 (4.32)	198 (3.77)	378 (4.02)	Physical files (46 files were partially abstracted)
Ontario	1311 (31.48)	1710 (32.59)	3021 (32.09)	Physical files and electronic database (data for 681 cases from 2017 came from the electronic database only)
Quebec	534 (12.82)	537 (10.23)	1068 (11.34)	Physical files for most cases and electronic coroner reports for 426 cases from 2017
Newfoundland and Labrador	30 (0.72)	42 (0.8)	75 (0.79)	Physical files
New Brunswick	66 (1.58)	63 (1.2)	126 (1.34)	Physical files
Nova Scotia	84 (2.02)	99 (1.89)	183 (1.94)	Physical and electronic files
Prince Edward Island	15 (0.36)	15 (0.28)	30 (0.32)	Physical files
Yukon	Suppressed	Suppressed	21 (0.22)	Physical files
Northwest Territories	Suppressed	Suppressed	Suppressed	Physical files
Nunavut	Suppressed	Suppressed	Suppressed	Physical files

^a^To protect privacy, counts have been randomly rounded to base 3 and numbers <10 have been suppressed. As counts have been randomly rounded to base 3, they may not add up when summed.

^b^Data from British Columbia were available only for people who experienced accidental or undetermined acute toxicity deaths involving “street drugs” or pharmaceutical substances not prescribed to them. As such, data for people who experienced acute toxicity deaths by suicide or due solely to prescribed substances or alcohol were not available.

Due to COVID-19–related public health measures, the ability to abstract physical case files was limited, resulting in data being collected from electronic files or databases in 2 provinces. Altogether, 681 of the 1710 Ontario cases from 2017 (22.54% of all Ontario cases) were mapped from an electronic database, and 426 of the 537 Quebec cases from 2017 (39.89% of all Quebec cases) were abstracted from electronic coroner reports rather than physical files.

### Data Quality Indicators

#### Consistency of Data Collection

Baseline measurements for the fictitious CME test file were within the established threshold for 96% (25/26) of all abstractors, and IaRR measurements taken thereafter over the course of abstraction remained within that threshold for 96% (25/26) of all abstractors. Abstractors who did not meet this threshold received remedial training and were reassessed. All obtained satisfactory κ values through this process, allowing them to start or resume abstraction.

#### Availability of Data on Key Concepts

Information collected from CME files was available when (1) it was systematically collected during death investigations, (2) it was collected during death investigations but not systematically, or (3) it was not specifically sought by the CME during death investigations but was serendipitously captured in the case file. As investigation protocols, forms, and records available to CMEs varied by jurisdiction, the availability of information also varied. Where the data source was an electronic file or database, less information was often available, except for electronic files from Saskatchewan, which included scanned copies of all paper documents in the case file.

Core study variables had good availability overall and across data sources (Table 3). Using a combination of postal codes and municipalities, the study was able to collect the census subdivision of residence for 95.44% (n=8985) of people who died during the study period. Full toxicology report results were available for 90.36% (n=8586) of people who died but were unavailable for 434 (4.61% of all people who died) Ontario 2017 cases that were mapped from an electronic database where only partial toxicology results were available. Toxicology information was also unavailable in instances where there was decomposition of the person before they were found (<1% of cases), a prolonged hospital stay before death (<1% of cases), or their hospital admission blood sample was not available (<1% of cases).

**Table 3:** Availability of information for core variables and key concepts among 9414 CME^a^ case files for our national chart review study of substance-related acute toxicity deaths.

Concept		Cases with unknown or unavailable variables, n (%)		Data sources with missing information for >90% of records^b,c^		
**Core variables**						
	Age		0 (0)	None		
	Sex		0 (0)	None		
	Manner of death		0 (0)	None		
	Date of death		0 (0)	None		
	Residence census subdivision		429 (4.56)	None		
	Residence postal code		2127 (22.59)	Quebec electronic files		
	Residence location type		1011 (10.74)	None		
	Acute toxicity event census subdivision		1218 (12.94)	None		
	Acute toxicity event postal code		1797 (19.09)	Quebec electronic files		
	Acute toxicity event location type		678 (7.2)	None		
	Death census subdivision		129 (1.37)	None		
	Death postal code		1581 (16.79)	Quebec electronic files		
	Death location type		69 (0.73)	None		
	Toxicology report available		828 (8.8)	None		
	Specific substances contributing to death		852 (9.05)	None		
	Classes of substances contributing to death		792 (8.41)	None		
**Key concepts**						
	Race		5304 (56.34)	Partially abstracted Manitoba cases, Quebec electronic files, and Nova Scotia		
	Ethnicity		8520 (90.5)	Alberta, Manitoba, Ontario electronic database, Quebec, New Brunswick, Nova Scotia, Prince Edward Island, Newfoundland and Labrador, and Yukon		
	Who the person who died lived with		4722 (50.16)	British Columbia, Ontario electronic database, and Prince Edward Island		
	Education		9012 (95.73)	British Columbia, Alberta, Saskatchewan, Manitoba, Ontario, Quebec electronic files, New Brunswick, Prince Edward Island, and Yukon		
	Income source		4572 (48.57)	British Columbia and Prince Edward Island		
	Occupation classification		7050 (74.89)	Saskatchewan, Quebec electronic files, and Prince Edward Island		
	Industry classification		7419 (78.81)	Saskatchewan, Quebec electronic files, and Prince Edward Island		
	Potentially traumatic events		5424 (57.62)	Prince Edward Island		
	Had an accessible family physician		6510 (69.15)	British Columbia and Prince Edward Island		
	Contact with the health system in the year before death		2124 (22.56)	None		
	Medical history		2775 (29.48)	None		
	Mental health history		2322 (24.67)	None		
	Prescription history		4536 (48.18)	British Columbia		
	History of substance use		1443 (15.33)	None		
	Known substances used		3174 (39.15)	British Columbia		
	Frequency of substance use (excluding alcohol) in the year before death		3114 (44.22)	Partially abstracted Manitoba cases, Ontario electronic database, Prince Edward Island, and Yukon		
	Substance use in the presence of others before the acute toxicity event		4011 (42.61)	Ontario electronic files		
	Witnesses to the acute toxicity event		3087 (32.79)	British Columbia		
	Apparent mode of substance use		5100 (54.17)	Partially abstracted Manitoba cases and Ontario electronic database		
	Presence of substances at the scene		4377 (46.49)	British Columbia		
	Tools of substance use at the scene		4275 (45.41)	British Columbia		
	Actions taken by witnesses		2544 (40.95)	None		
	First responder actions		888 (23.44)	British Columbia		
	Whether naloxone was administered		1713 (45.22)	Prince Edward Island		

^a^CME: coroner and medical examiner.

^b^This table excludes data from Nunavut and the Northwest Territories due to small numbers.

^c^Denominators for specific variables may not be the sum of all cases due to skip patterns in the data collection tool.

In general, information on socioeconomic variables (including education, occupation, and industry), potentially traumatic life events, and socially constructed identities (such as gender [not shown], ethnicity, and race) were often unavailable. Conversely, variables related to the person’s health, history of substance use, and events surrounding the acute toxicity event were available for most records. Some exceptions include whether the person had an accessible family physician (missing: n=6510, 69.15%), the apparent mode of substance use at the time of the acute toxicity event leading to death (missing: n=5100, 54.17%), and whether naloxone was administered (missing: n=1713, 45.22%).

## Discussion

### Principal Findings

As Canada continues to grapple with an ongoing overdose crisis, there is an increased need for high-quality data that can support clinicians, public health experts, and advocates in their efforts to refine interventions. This can be supported by relevant, comparable, and timely CME data.

Our study confirms that CME files are a rich source of information that is highly relevant to public health practitioners. Work has begun on knowledge translation products [46-52], including a summary report of findings from this study that provides insight into national trends over time and by geography, sociodemographic factors, health and substance use history, the circumstances of the acute toxicity event, and substances involved [48]. Improving the usefulness of CME files as sources for acute toxicity mortality data will depend upon how the Canadian CCCME offices meet 3 broad challenges. First, CMEs do not primarily collect data for the purpose of public health intervention but rather to meet their investigation mandate needs. As illustrated in Table 3, the availability of variables of public health interest varies across provinces and territories. Second, CME offices have different approaches to the collection, storage, and transfer of data. With respect to data infrastructure, some offices have electronic systems that can be readily searched, and some keep mostly paper-based files. Most of the electronic systems that do exist are not compatible with each other. Third, as CMEs use classification systems and terminology that suit the needs of local internal and external stakeholders, systems and terms may not be comparable across provinces and territories, necessitating resource-intensive studies such as the one described here to allow for comparable data.

Recognizing some of these challenges and opportunities, the PHAC, all 13 Canadian CCCME offices, and Statistics Canada established the CCCME and Public Health Collaborative in 2021, with a mandate to support the development of common approaches to death investigations and data infrastructure requirements. The collaborative has identified substance-related acute toxicity as one of its prioritized causes of death and will consider the knowledge gaps and lessons learned from this study in its development of core and minimum data elements to improve the comparability, usefulness, and accessibility of national mortality data. Increased knowledge transfer and collaboration across public health and CCCME jurisdictions in the development and implementation of common practices in death investigations will enhance the availability of timely and comparable data for public health use.

The authors are aware of how critical timeliness is to public health practitioners and community members who work on interventions. This study took longer than anticipated, partly due to challenges encountered during the global COVID-19 pandemic as well as navigating legislative and administrative requirements aimed at ensuring good data governance across multiple departments within 14 federal, provincial, and territorial governments. We anticipate that future similar national studies and surveillance activities can now occur more efficiently using the pathways created for this study. While data collected by this study may no longer reflect current trends in acute toxicity deaths, they provide an important baseline early in Canada’s overdose crisis that can be used to measure future progress.

### Limitations

Although efforts were made to identify all CME files that met the study case definition, it is possible that some people who died of acute toxicity were not included in this study if (1) their death was not reported to the CME office or (2) their death was not identified as a potential case. Systematic differences in study data sources (Table 2); how data were abstracted; and data availability associated with differences in death investigation processes, death classification methods, and toxicology testing methods (Table 3) may result in an underestimation of burden and could bias findings. Extensive standardized training and documentation were provided to abstractors, all of whom underwent IaRR assessments throughout data collection. Nonetheless, as data from each case file was collected from a single abstractor, error and bias may have resulted from differences in how abstractors assessed whether cases met eligibility requirements and abstracted data.

As most variables collected by our study had a nontrivial amount of missing data, study findings will be reported as minimum counts, proportions, and mortality rate estimates that likely underestimate the true population prevalence of reported characteristics. Results from variables with a high percentage of missing data should be interpreted with caution, particularly because the distribution of values where the information was known may not be the same as the distribution of values where information was unknown. The lack of information on demographic and socioeconomic variables and socially constructed identities (eg, gender) will hinder our ability to apply a sex- and gender-based analysis plus approach to our analyses, although efforts will be made to describe the distribution of people who died by sex as well as area-level characteristics where possible.

### Conclusions

This study provides the most detailed national information on substance-related acute toxicity deaths in Canada to date. It is anticipated that results will allow for comparisons across provinces and territories, provide evidence to inform programs and policies at all levels, inform the expansion of national surveillance activities on substance-related harms, supply evidence to inform the development of common approaches to medicolegal death investigations and routine postmortem testing, and serve as a platform for future research activities. It also provides a baseline of acute toxicity mortality before the COVID-19 pandemic for assessing the evolution of the overdose crisis. Finally, the partnerships and processes initiated by this study have contributed to fruitful new collaborations between Canada’s public health and CME communities.

## Appendix

Multimedia Appendix 1 Variable list.

Multimedia Appendix 2 Database screenshots.

## Abbreviations

CCCME: chief coroner and chief medical examiner
CME: coroner and medical examiner
IaRR: intrarater reliability
PHAC: Public Health Agency of Canada

## Data Availability

The study dataset is not publicly available due to provisions in the data-sharing agreements with provincial and territorial data providers. The corresponding author can assist in directing inquiries about data access to the original data providers.
