# Supplementary material for: Substance-Related Acute Toxicity Deaths in Canada From 2016 to 2017: Protocol for a Retrospective Chart Review Study of Coroner and Medical Examiner Files
Source: JMIR Public Health Surveill. 2025 Apr 10;11:e49981. doi: 10.2196/49981 (PMC12022517; doi:10.2196/49981)
Supplement: Multimedia Appendix 1 [file publichealth_v11i1e49981_app1.docx]

Substance-related acute toxicity deaths in Canada from 2016 to 2017: A protocol for a retrospective chart review study of coroner and medical examiner files

# Appendix 1: Variables collected for our national chart review study of substance-related acute toxicity deaths.

Approximately 1,300 variables were collected for our national chart review study of substance-related acute toxicity deaths and numerous derived variables were created during the data preparation stage. To decrease the size of this variable list, variables collected for a similar concept have been collapsed in this table. For example, although date of death appears as a single variable in this list, it was actually collected as three different variables: day of death, month of death, and year of death.

Note that the category titles included in this variable list were those used for the data collection tool and that some of these variables were recategorized during the data preparation and analysis phases of the study. For example, although ketamine appears in the "hallucinogens and stimulants" category here, it was moved to "other substance types" category during data preparation.

## Administrative variables

Table 1. Administrative variables collected for our national chart review study of substance-related acute toxicity deaths.

| No. | Variable | Description |
| --- | --- | --- |
| 1.1 | Case definition met | Indicates if the person who died met the study case definition. |
| 1.2 | Study ID | A unique 8-digit identifier assigned to the case by the database. |
| 1.3 | Abstractor ID | A two-digit identifier unique to each data abstractor in the study. |
| 1.4 | Date of death | The day, month, and year on which the person died, was pronounced dead, or was found dead. |
| 1.5 | Day of the week of death | The specific day of the week when the person died, was pronounced dead, or the estimated day of death. |
| 1.6 | Province or territory of death | The province or territory where the death occurred or the body of the person who died was found. |
| 1.7 | C/ME ID | The identification number associated with the case in the information system of the reporting C/ME office. |
| 1.8 | Collection date | The date that case data was abstracted by the study data abstractor. |
| 1.9 | File status | The status of the C/ME’s file at the time of data abstraction. Options include:   - Open (active) = Cases where the C/ME investigation was in progress at the time of data abstraction. - Closed (completed) = Cases where the C/ME investigation was concluded and the C/ME had made their declaration. |
| 1.10 | Follow-up required | Indicates if any follow-up with the data provider or study team was required for the record. |
| 1.11 | Discard | Indicates if the case met the requirements of the case definition or should be discarded. Information on the reason why a case was discarded was also collected. |
| 1.12 | Lock the record from editing | Allows the user to lock the record from editing. |

## Case information

Table 2. Case information variables collected for our national chart review study of substance-related acute toxicity deaths.

| No. | Variable name | Description |
| --- | --- | --- |
| 2.1 | Province/territory of residence | The province or territory of residence of the person who died. |
| 2.2 | Municipality of residence | The Statistics Canada census subdivision of residence of the person who died. Census subdivision is the general term for municipalities (as determined by provincial or territorial legislation) or areas treated as municipal equivalents for statistical purposes (e.g., Indian reserves, Indian settlements and unorganized territories) [1]. |
| 2.3 | Postal code of residence | The postal code of residence of the person who died. |
| 2.4 | Community size and metropolitan influence zone (CSizeMIZ) | Describes the community population size of the person who died using Statistics Canada categories [2], based on their postal code or municipality of residence. CSizeMIZ classifies urban areas based on the 2016 Census population size of each census metropolitan area (CMA; population of at least 100,000 residents) or census agglomeration (CA; population of at least 10,000 residents). Rural areas and remote areas outside of CMAs and CAs are classified by metropolitan influence zones according to the degree of influence of CMAs and CAs (strong, moderate, weak, or no influence), based on the percentage of the population who commute to work in a CMA or CA core. Urban community population size values include 1,500,000 or more residents, 500,000 to 1,499,999 residents, 100,000 to 499,999 residents, and 10,000 to 99,999 residents. Rural community population size values include strong MIZ, moderate MIZ, and weak or no MIZ. |
| 2.5 | Community remoteness | Measures the geographic proximity of the census subdivision of residence of the person who died to population centres, according to a given travel radius and population size [2]. Community remoteness is measured using a variable derived from the Index of Remoteness (RI), which assigns relative remoteness values to almost all Canadian census subdivisions on a scale of 0 to 1, where 0 is the most accessible community and 1 is the least accessible (or remote) community. The continuous RI values are classified into five discrete categories including easily accessible areas (RI: <0.1500), accessible areas (RI: 0.1500 to 0.2888), less accessible areas (RI: 0.2889 to 0.3898), remote areas (RI: 0.3899 to 0.5532), and very remote areas (RI: > 0.5532) [3]. |
| 2.6 | Neighbourhood-level indicators of deprivation | Describes neighbourhood-level indicators of deprivation based on the postal code of residence of the person who died using Statistic Canada’s Canadian Index of Multiple Deprivation (CIMD) [4]. The CIMD is a validated, geographically-based index of material and social deprivation that uses information from the 2016 Census and assigns each dissemination area in Canada a quintile ranking, where a value of 1 corresponds to dissemination areas with the lowest level of deprivation or concentration for that dimension and a value of 5 corresponds to dissemination areas with the highest level of deprivation or concentration. It consists of four composite dimensions, including:   - Residential instability: This dimension relates to changes in neighbourhood populations over time. It combines the proportion of dwellings that are apartment buildings, dwellings that are owned, persons living alone, the population who moved within the past five years, and the population that is married or common-law. - Economic dependency: This dimension relates to sources of income and labour force participation. This dimension combines the proportion of the population aged 65 and older, the proportion of the population participating in the labour force, the ratio of employment to population, the dependency ratio (population aged 0 to 14 and aged 65 and older divided by population aged 15 to 64), and the proportion of the population receiving government transfer payments. - Ethno-cultural composition: This dimension combines the concentration of the population who self-identify as a visible minority, who are recent immigrants, who are foreign-born, and who do not have knowledge of either official language (English or French). - Situational vulnerability: This dimension relates to variations in housing, education, and demographic characteristics and combines the proportion of dwellings needing major repairs, the population that identifies as Indigenous, and the population aged 25 to 64 without a high school diploma. |
| 2.7 | Area-based neighbourhood income quintile after tax | Indicates the after-tax income quintile of the neighbourhood of the person who died using their postal code of residence and Statistic Canada’s Postal Code Conversion File Plus [2]. These quintiles are based on census metropolitan areas (population of at least 100,000 residents) and census agglomerations (population of at least 10,000 residents) to control for differences in the cost of living across Canada. |
| 2.8 | Age in years | The age in years of the person who died at the time of death. The estimated age of the case (as per the C/ME file) was also abstracted when the exact age was not available. |
| 2.9 | Age group A | The age group of the person who died based on their age at the time of death. Values include less than or equal to 19 years old, 20 to 29 years old, 30 to 39 years old, 40 to 49 years old, 50 to 59 years old, 60 to 69 years old, 70 years old or greater, or unknown. |

## Demographic and socioeconomic factors

Table 3. Demographic and socioeconomic variables collected for our national chart review study of substance-related acute toxicity deaths.

| No. | Variable name | Description |
| --- | --- | --- |
| 3.1 | Race | Describes the race categories that best describe the person who died, as per the C/ME file. Values for this variable were obtained from Ontario’s Data Standards for the Identification and Monitoring of Systemic Racism [5]. Options include:   - Black: African, African-Caribbean, or African-Canadian descent - East/Southeast Asian: Chinese, Korean, Japanese, Taiwanese descent; Filipino, Vietnamese, Cambodian, Thai, Indonesian, or other Southeast Asian descent - Indigenous: First Nations, Métis, or Inuit descent - Latino: Latin American or Hispanic descent - Middle Eastern: Arab, Persian, or West Asian descent (e.g., Afghan, Egyptian, Iranian, Lebanese, Turkish, Kurdish, etc.) - South Asian: South Asian descent (e.g., East Indian, Pakistani, Bengladeshi, Sri-Lankan, Indo-Caribbean, etc.) - White: European descent - Other - Unknown |
| 3.2 | Ethnic origins | Describes if the person who died belongs to a specific ethnic group or population. Options included North American Indigenous origins, other North American origins, European origins, Caribbean origins, Latin, Central and South American origins, African origins, Asian origins, Oceania origins, other, and unknown. |
| 3.3 | Indigenous group | Describes the specific Indigenous group of the person who died as per the C/ME file. Options included:   - First Nations: The person who died self-identified as First Nations. - Métis: The person who died self-identified as Métis. - Inuit: The person who died self-identified as Inuit. - Indigenous (identity not otherwise specified): The person who died self-identified as Indigenous but further disaggregation into First Nations, Métis, or Inuit status is not possible. - Multiple Indigenous identities: The person who died self-identified as having multiple Indigenous identities. - Non-Indigenous: The person who died did not self-identify as Indigenous. - Unknown if Indigenous: It is unknown whether the person who died self-identified as Indigenous. |
| 3.4 | On reserve or land claim region | Indicates if the address of residence of the person who died was on a First Nations reserve, land claim region, or a community governed by land-claim decisions or self-government agreements. |
| 3.5 | Immigration status or country of birth | Any information in the C/ME file regarding the immigration status or country of birth of the person who died. |
| 3.6 | Sex | The biological sex of the person who died according to the C/ME file. |
| 3.7 | Gender | The gender of the person who died as described in the C/ME file. |
| 3.8 | Sexual orientation | The sexual orientation with whom the person who died was emotionally, mentally, and physically attracted to, based on gender in relation to the case.   - Heterosexual: Attracted emotionally and/or sexually to persons of the opposite gender. - Gay or lesbian: Attracted emotionally and/or sexually to persons of the same gender. - Bisexual: Attracted emotionally and/or sexually to two or more genders. - Other: Please specify. - Unknown: The sexual orientation of the case is unknown. |
| 3.9 | Apparent sex or gender of partner | If the person who died was in a relationship at the time of death or if they had recently been in a relationship, the apparent sex or gender of their partner (if mentioned in their file). |
| 3.10 | Relationship status | The relationship status of the case at time of death. Options include:   - Common-law: Lived in a conjugal relationship outside marriage, but was not legally married to that person, as defined by:   - Cohabitating for at least one year.   - Cohabitating and were together the parents of a child.   - Entered into a cohabitation agreement. - Married: A person who was married and had not separated or obtained a divorce, and whose spouse was living. - Other partner: A person who has a partner that was not described as a common-law or marital spouse (e.g. boyfriend, girlfriend, partner). - Divorced: A person who had obtained a legal divorce and who had not remarried. Persons living in a common-law relationship or described as having an "other partner" are not included in this category. - Separated: A person who was married but who was no longer living with their spouse (for any reason other than illness, work, or school) and who had not obtained a divorce (that is, they were still legally married). Persons living in a common-law relationship are not included in this category. - Single: A person who had never married or whose marriage had been annulled, and who had not remarried. Persons living in a common-law relationship are not included in this category. - Widowed: A person who had lost their spouse through death and who had not remarried. Persons living in a common-law relationship are not included in this category. - Other: The relationship status of the case does not align with any of the categories as described above. - Unknown: The relationship status of the case is unknown. |
| 3.11 | Children of the deceased | Indicates if the person who died had adult and minor children. Options include no children; yes, only children 18 years or older; yes, including children under 18 years old; yes, but unknown if any child was less than 18 years old; or unknown. |
| 3.12 | Care of underage children (<18 years) | Provides information on who was taking care of the child(ren) of the person wo died at time of their death if they had children under 18 years of age. Options include under the full-time care of the person wo died; under the part-time care of the person wo died; under the care of family members; under the care of child welfare services; other; or unknown. |
| 3.13 | Living situation | Describes who the person who died lived with in their place of usual residence at the time of their death. Options include alone; with parents; with family (spouse, common-law partner, or children); with a partner (not common-law or unknown if common-law); with relatives (other than parents, spouse, common-law partner, children); with friends or roommates; with others; or unknown. |
| 3.14 | Living arrangements | The type of residence that the person who died lived in primarily at the time of death.   - Private dwelling: A separate set of living quarters designed (or converted) for human habitation. Must include a source of heat or power and must be an enclosed space that provides shelter or protection from the elements. This includes owning or renting a residence or renting a room or secondary suit within a residence and staying with friends or family. - Retirement home (including senior residences): Residences for older persons that provide support services and supervision of residents. - Long-term care facility or nursing home: Home-based health care facilities designed for adults who need access to on-site 24-hour nursing care, frequent assistance with activities of daily living (i.e., eating, bathing, toileting, etc.) and monitoring for safety or well-being. They are also known as nursing homes, charitable homes, or municipal homes for the aged. - Hotel or motel - Mental health facility: An institution or establishment providing residential mental health care. - Substance use/addictions treatment facility or harm reduction residence: An institution or establishment providing residential treatment for substance use and treatment, including harm reduction residences or ‘recovery homes’. - Hospital - Correctional facility or police custody: May include federal correctional institutions, provincial and territorial custodial facilities, young offender facilities, jails, and police lock-up facilities. - Supportive or transitional housing (e.g., a group home or halfway home): Supportive housing is generally long-term accommodation that provides a varying range of supportive services depending on the needs of residents. It includes accommodation for people facing barriers to housing or living independently (for example, people with a mental illness, who use substances, or who have lost autonomy in older age). Transitional housing is intended to maximize independence and support the development of social, vocational, recreational, and life skills. These homes can be used to house children and youth, adults or seniors with chronic disabilities, persons seeking recovery from substance use, or persons re-integrating with the community following incarceration. It is considered an intermediate step between emergency shelter and supportive housing and has its limits on how long a person or family can stay. - Experiencing homelessness: Describes the situation of a person without stable, safe, or appropriate housing, or the immediate means or ability to acquire it [6]. This includes people living unsheltered on the street, staying in emergency shelters, or temporarily accommodated by couch surfing or staying with friends or family. It also includes people at immediate risk of homelessness because of job loss or eviction by a property owner, for example. - Other - Unknown |
| 3.15 | Recent release from an institution | Indicates if the person who died had recently been released from an institution and the type of institution from which they had been released. Options include a correctional facility, remand centre, or young offender centre; hospital; mental health facility; long-term residential health facility (e.g., nursing home); other health facility; supervised residential facility related to alcohol or substance use treatment; supervised residential facilities not related to alcohol or substance use treatment; type of institution unspecified; other; or unknown or no evidence of recent release. |
| 3.16 | Time since release from institution | Describes the time since release from an institution up to a year preceding death. |
| 3.17 | Recently moved from another place | Describes whether the case had recently moved (in the six months preceding death) from another place, city, province, territory, or country and the reason why. This excludes any recent release from an institution. |
| 3.18 | Educational level | The highest educational qualification achieved by the person who died. Options include less than their high school diploma; high school diploma; some level of college education or college diploma; some level of university education or greater; or unknown. |
| 3.19 | Income source and employment at the time of death | The income source and/or employment status of the person who died at the time of death (used to generate income). Options include (multiple selections are possible) employed full-time; employed part-time; employed, unknown if full-time or part-time; seasonal worker; unemployed; retired; student (full- or part-time); social assistance program; disability supports; personal or family responsibilities; illegal sources of income; permanently unable to work due to physical or mental disability; other; or unknown. |
| 3.20 | Transition in employment status/occupation/income source at time of death | Indicates whether the person who died had been experiencing a transition or change in their employment status/occupation/income source at the time of death. |
| 3.21 | Occupation classification | The classification of the occupation of the person who died at the time of death *or* the previous occupation if the person was unemployed or retired at time of death. The occupation classification represents the nature of the work that the person carried out. This is assigned by the data abstractor using the information available and is based on Statistics Canada’s National Occupational Classification [7]. Options include (multiple selections are possible) management occupation; business, finance, and administration occupation; natural and applied sciences and related occupation; health occupation; occupation in education, law, social, community and government services; occupation in art, culture, recreation and sport; sales and services occupation; trades, transport and equipment operators and related occupation; natural resources, agriculture and related production occupation; occupation in manufacturing and utilities; other; and unknown. |
| 3.22 | Industry classification | The classification of the industry of the employment of the person who died at the time of death *or* the previous industry if they were unemployed or retired at the time of death. The industry classification represents the broader type of activity carried out at the place of work (not the person’s specific role at their place of work). This is assigned by the data abstractor using the information available and is based on the classifications and definitions provided by Canadian Industry Statistics [8]. Options include (multiple selections are possible) accommodation and food services; administrative and support, waste management, and remediation services; agriculture, forestry, fishing, and hunting; arts, entertainment, and recreation; construction; educational services; finance and insurance; health care and social assistance; information and cultural industries; management of companies and enterprises; manufacturing; mining, quarrying, and oil and gas extraction; professional, scientific, and technical services; public administration; real estate and rental and leasing; retail trade; transportation and warehousing; utilities; wholesale trade; other services (except public administration); and unknown. |
| 3.23 | Ever served in military | Indicates if the person who died had ever provided service in any division of the armed forces. |
| 3.24 | Currently a veteran | Indicates if the person who died was currently a veteran. |
| 3.25 | History of correctional involvement | Indicates if the person who died had previously spent time incarcerated or in detention in a correctional facility, which may include a provincial correctional facility, remand centre, or federal prison. |
| 3.26 | Time of most recent incarceration | Describes the time interval between the date of release from their last detention and death is collected. Options include detained at time of death, released up to one week before death, released from 1 week to 1 month before death, released between > 1 month and 1 year, released > 1 year before death, or unknown. |
| 3.27 | Place of most recent incarceration | Describes the detention facilities where the case was most recently held. Options include a federal correctional facility; provincial/territorial correctional facility; remand centre; young offender centre; unknown type of facility; other; or unknown. |
| 3.28 | Potentially traumatic life events | Describes any mentioned life experiences that the person who died may have experienced prior to death, potentially serving as a source of stress or trauma. Trauma results from "an event, series of events, or set of circumstances that is experienced by an individual as physically or emotionally harmful or life threatening and that has lasting adverse effects on the individual's functioning and mental, physical, social, emotional, or spiritual well-being."[9] There is no restriction on the time period in which these life events could have occurred, though data abstractors could indicate whether any of the events occurred within two weeks of the death. Potentially traumatic events might include a health problem of a family member or relative, intimate partner problems (e.g., divorce, discord), other relationship problems (e.g., family argument), job problems (e.g., layoff, pressure), school problems (e.g., problems with grades, bullying), financial problems (e.g., debts, bankruptcy), recent death by suicide of friends or family members, other death of friends or family members, criminal legal problems (e.g., arrest, jail, court), other legal problems (e.g., custody dispute, civil law), perpetrator of interpersonal violence, victim of interpersonal violence, victim of child abuse, foster care experiences, residential school experiences, experience of sexual abuse, or experience of physical abuse or assault. |

## Drug and medical history

Table 4. Drug and medical history variables collected for our national chart review study of substance-related acute toxicity deaths.

| No. | Variable name | Description |
| --- | --- | --- |
| 4.1 | Had an accessible family doctor to receive regular care from, if needed | Indicates whether the person who died had an accessible family doctor or primary care physician from which they could receive regular care, if needed. Any record available in the file can be consulted to determine whether they had a family doctor. Data abstractors were instructed to assume that the physician was accessible so long as there was no evidence that the person who died had difficulty accessing them for health care services. |
| 4.2 | Did the decedent have any contact with health services in the preceding year? | Indicates if the person who died had any contact with health care services in the year preceding death (excluding any related to the acute toxicity event that resulted in their death). |
| 4.3 | Indicate type of contact with health services | For those who had contact with health services in the year preceding death, indicates if they received outpatient care (contact with health services that did not require inpatient admission) or if they were admitted as an inpatient. |
| 4.4 | Outpatient treatment type | For those who received outpatient care in the year preceding death, indicates the type of outpatient care sought and/or received. Options include EMS, emergency department, care from a general or a nurse practitioner, other, or unknown. |
| 4.5 | Inpatient admission type | For those admitted as an inpatient in the year preceding death, indicates if the person who died was admitted to a hospital or another place. |
| 4.6 | Indicate what services or conditions the case sought assistance for | Indicates the type of health conditions for which care was sought in the year preceding death. Options include (multiple selections allowed) acute injury; pain-related; acute toxicity event; substance use or addictions; mental health; surgery; other; or unknown. |
| 4.7 | Support offered on discharge | If the health condition for which care was sought in the year preceding death was an acute toxicity event or substance use or addictions, describes whether the person who died was referred to a drug-support program while getting discharged from or leaving the health care facility. Options include (multiple selections allowed) no support provided; yes, referral to a substance use program; yes, a take home naloxone kit was provided upon discharge; other; or unknown. |
| 4.8 | Negative experiences with or difficulties accessing the health care system | Indicates if the person who died had experienced any known barriers to care such as negative experiences (e.g., stigma) with the health care system or difficulties accessing the health care system/services. |
| 4.9 | Medical history | Describes the current or past medical conditions experienced by the person who died, as described in the C/ME file. Data sources may include medical records or witness statements (e.g., from friends and family), therefore these may not be clinical diagnoses. Options include (multiple selections allowed) the file states that the person who died had no history of medical issues; back pain; other pain disorder or chronic pain; acute pain; pain, unspecified; chronic lung disease; chronic liver disease; injury; heart disease; sleep apnea; chronic kidney disease; endocarditis; hepatitis B; hepatitis C; HIV or AIDS; diabetes; long-term (>90 days) treatment with opioid(s) for pain; cellulitis; pregnant or recently gave birth; past surgery; syphilis; chronic obstructive pulmonary disease (COPD); asthma; migraine; obesity; other; or unknown. |
| 4.10 | Mental health history | Describes the mental health history of the person who died, as described in the C/ME file. Data sources may include medical records or witness statements (e.g., from friends and family), therefore these may not be clinical diagnoses. Options include (multiple selections allowed) that the file states that the person who died had no history of mental health issues; depression (may be depressive symptoms or clinical depression); bipolar disorder; evidence of suicidal ideation or attempt; schizophrenia; anxiety disorder; post-traumatic stress disorder; eating disorder; alcohol use disorder; substance use disorder; personality disorder; inpatient mental health treatment; attention deficit/hyperactivity disorder (ADHD); obsessive-compulsive disorder; other; or unknown. |
| 4.11 | Current or recent prescribed medication | Describes any medication that was prescribed to the person who died up to six months before death, as described in the C/ME file. Data sources may include medical records or scene evidence. Options include (multiple selections allowed) no medication prescribed; opioids; cannabinoids; benzodiazepines; muscle relaxants; antidepressants; antipsychotics; prescription stimulants; opioid agonist therapy; gabapentinoids; other; or unknown. |
| 4.12 | Opioid medication denied or reduced | Describes if the opioid prescription of the person who died had been denied or reduced in the six months prior to death and the reason why. |

## Substance use history

Table 5. Substance use history variables collected for our national chart review study of substance-related acute toxicity deaths.

| No. | Variable name | Description |
| --- | --- | --- |
| 5.1 | History of substance use | Specifies whether there is any evidence in the C/ME file of the person who died having a history of substance use (outside of the fatal acute toxicity event) that involved use of:   - Non-pharmaceutical substances (including those described as "illegal" or "illicit" in coroner and medical examiner files) - Diverted pharmaceuticals or pharmaceuticals used not as prescribed (not as prescribed does not include the concurrent use of alcohol with benzodiazepines, opioids, or other medications where alcohol use is not recommended) - Over-the-counter medications that were known to be taken not as indicated - Inhalants (including solvents, aerosols, gases, and nitrites) - Alcohol |
| 5.2 | Known substances used | Describes known substances that were associated with a history of substance use for the person who died. Options include alcohol, amphetamine, benzodiazepine, buprenorphine, codeine, cocaine, diacetylmorphine (heroin), fentanyl, hydromorphone, methadone, morphine, oxycodone, other prescription stimulants, suboxone, cannabis or cannabinoids, gabapentinoids, methamphetamine, MDMA (ecstasy), opioids (non-specific), non-opioids (non-specific), or other. |
| 5.3 | Frequency of substance use (excluding alcohol) in the year preceding death | Describes the pattern of substance use (excluding alcohol) of the person who died in the year preceding their death. Options include:   - No current known use: Refers to people with a history of substance use, but where it is reported in the case file that there was no known use in the year preceding death. - Less than chronic use: Refers to any use that is less than daily or almost daily (e.g., use that may be described as occasional, recreational, just on weekends, etc.). - Chronic use: Refers to daily or almost daily use. - Unclear if less than chronic versus chronic use: A frequency is provided but there is insufficient information to distinguish between these two categories. - Unknown frequency of use: Refers to people with a history of substance use but who had no information available regarding the frequency of use. |
| 5.4 | Frequency of alcohol use in the year preceding death | Describes the pattern of alcohol use of the person who died in the year preceding their death. Options include:   - No current known use: Refers to people with a history of alcohol use, but where it is reported in the case file that there was no known use in the year preceding death. - Less than chronic use: Refers to any use that is less than daily or almost daily (e.g., use that may be described as occasional, recreational, just on weekends, etc.). - Chronic use: Refers to daily or almost daily use. - Unclear if less than chronic versus chronic use: A frequency is provided but there is insufficient information to distinguish between these two categories. - Unknown frequency of use: Refers to people with a history of alcohol use but who had no information available regarding the frequency of use. |
| 5.5 | Mention of a change in frequency of use (within preceding year) | Provides information on whether the person who died was known to have changed the frequency of their substance use in the period preceding the death. Options include no change mentioned, an increase, or a decrease. |
| 5.6 | Proximity to death of a change in frequency of use (within preceding year) | If there was any mention in the case file of a change in frequency in substance use in the year preceding death, indicates how long before the death it occurred. If multiple changes in frequency are mentioned, the change that was closest in proximity to the death was recorded. Options include less than 2 weeks, 2 weeks to less than 1 month, 1 month to 3 months, more than 3 months to 1 year, or unspecified. |
| 5.7 | Mention of any experience of chronic substance use | Indicates if the C/ME file of the person who died mentions chronic substance use at any point in their lifetime. |
| 5.8 | Evidence of potential social impact as a result of substance use | Provides information on possible negative life events and experiences of the person who died because of substance use. To be included here, it must be clearly stated in the C/ME file that the occurrence of these events was a result of substance use. If a file stated that a person experienced these events but it was not explicitly stated that they were the result of substance use, it was not included in this section. Options include removal of children from care, job loss, correctional involvement (due to a substance-use related offence), negative experiences of friends or family, housing instability, other, or none of the above are mentioned in the file. |
| 5.9 | Acute toxicity event resulting in death occurred after a period of discontinued substance use | Indicates if the acute toxicity event resulting in death occurred after a period of discontinued substance use. |
| 5.10 | Evidence of any injection drug use | Indicates if there is evidence in the C/ME file of any injection drug use. This includes any evidence that the apparent mode of drug use with respect to the acute toxicity event that resulted in death was injection or evidence that the person had used injection drugs at any point in their life. |
| 5.11 | Trying to get into treatment | Indicates if the person who died was trying to get into a treatment program at the time of their death. Since people may seek out more than one form of treatment, enrolment in a treatment program at the time of death did not necessarily indicate that they were not trying to get into another program. |
| 5.12 | In or receiving treatment | Indicates if the person who died was in or receiving treatment for a substance use disorder or problematic substance use at the time of their death. |
| 5.13 | Ever received treatment for substance use disorder or problematic substance use | Indicates if the person who died had ever received treatment for a substance use disorder or problematic substance use. |
| 5.14 | Substance use disorder treatment type | Describes what types of treatment the person who died had ever received for a substance use disorder or problematic substance use. Options include:   - Brief detox: A safe place to sleep for individuals under the influence of alcohol or drugs where they can be supervised until no longer intoxicated from their alcohol or substance use. - Residential treatment - Suboxone therapy - Methadone therapy - Injectable diacetylmorphine - Injectable hydromorphone - Psychosocial treatment: These can include but are not limited to psychotherapy (e.g., cognitive behavioural therapy, relapse prevention), counselling, contingency management, and community reinforcement approach. - Pharmacological treatment - Slow acting morphine therapy - Opioid use treatment, unspecified - Other - Other pharmacological treatment - Unknown pharmacological treatment - Unknown |
| 5.15 | Discharged from (or home visiting from) residential treatment within about 30 days of death | Among people who died with a history of receiving residential treatment, describes if they had been discharged from (or home visiting from) residential treatment within about 30 days of death. |
| 5.16 | Methadone therapy | For those who had ever received methadone therapy, describes when they had last received it. Options include no history within the past year; yes, currently receiving at the time of death; not currently receiving it but previously received within the past year; or unknown. |
| 5.17 | Suboxone therapy | For those who had ever received suboxone therapy, describes when they had last received it. Options include no history within the past year; yes, currently receiving at the time of death; not currently receiving it but previously received within the past year; or unknown. |
| 5.18 | Injectable diacetylmorphine therapy | For those who had ever received injectable diacetylmorphine therapy, describes when they had last received it. Options include no history within the past year; yes, currently receiving at the time of death; not currently receiving it but previously received within the past year; or unknown. |
| 5.19 | Injectable hydromorphone therapy | For those who had ever received injectable hydromorphone therapy, describes when they had last received it. Options include no history within the past year; yes, currently receiving at the time of death; not currently receiving it but previously received within the past year; or unknown. |
| 5.20 | Slow acting morphine therapy | For those who had ever received slow acting morphine therapy, describes when they had last received it. Options include no history within the past year; yes, currently receiving at the time of death; not currently receiving it but previously received within the past year; or unknown. |
| 5.21 | Brief detox | For those who had ever undergone a brief detox, describes when they had last undergone it. Options include no history within the past year; yes, currently undergoing at the time of death; not currently undergoing it but previously done so within the past year; or unknown. |
| 5.22 | Psychosocial treatment | For those who had ever received psychosocial treatment, describes when they had last received it. Options include no history within the past year; yes, currently receiving at the time of death; not currently receiving it but previously received within the past year; or unknown. |
| 5.23 | Residential treatment | For those who had ever received residential treatment, describes when they had last received it. Options include no history within the past year; yes, currently receiving at the time of death; not currently receiving it but previously received within the past year; or unknown. |
| 5.24 | Opioid treatment, unspecified | For those who had ever received unspecified opioid treatment, describes when they had last received it. Options include no history within the past year; yes, currently receiving at the time of death; not currently receiving it but previously received within the past year; or unknown. |
| 5.25 | Evidence of non-fatal acute toxicity event | Indicates if there is any anecdotal evidence that the person who died had previously experienced a non-fatal acute toxicity event. |
| 5.26 | Time since most recent non-fatal acute toxicity event | If the person who died was known to have previously experienced a non-fatal acute toxicity event, indicates how long ago the most recent event took place. Options include < 1 week, ≥ 1 week but < 1 month, ≥ 1 month but < 1 year, ≥ 1 year, or unknown. |
| 5.27 | Recent non-fatal acute toxicity event specified | If the person who died was known to have previously experienced a non-fatal acute toxicity event, this variable provides a brief summary of the most recent non-fatal acute toxicity event, including the time, place, and substances involved. |

## Circumstances of death and recommendations

Table 6. Circumstances of death and recommendation variables collected for our national chart review study of substance-related acute toxicity deaths.

| No. | Variable name | Description |
| --- | --- | --- |
| 6.1 | Death location same as residence location | Indicates if the place of death location was the place of residence of the person who died. |
| 6.2 | Postal code of death | The postal code of the place of death. |
| 6.3 | Municipality of death | The Statistics Canada census subdivision where the person died. Census subdivision is the general term for municipalities (as determined by provincial/territorial legislation) or areas treated as municipal equivalents for statistical purposes (e.g., Indian reserves, Indian settlements and unorganized territories) [1]. |
| 6.4 | Death took place on Indigenous reserve or land claim region | Indicates if the death took place on a First Nations reserve, land claim region, or a community governed by land-claim decisions or self-government agreements. |
| 6.5 | Place of death | The place where the death took place. Options include:   - Personal residence setting (i.e., the home address of the person who died, including multi-tenant housing such as ‘rooming houses’) - Home of another person (a shared home or a friend or relative’s home) - Supportive or transitional housing (e.g., a group home or halfway home): Supportive housing is generally long-term accommodation that provides a varying range of supportive services depending on the needs of residents. It includes accommodation for people facing barriers to housing or living independently (for example, people with a mental illness, who use substances, or who have lost autonomy in older age). Transitional housing is intended to maximize independence and support the development of social, vocational, recreational, and life skills. These homes can be used to house children and youth, adults or seniors with chronic disabilities, persons seeking recovery from substance use, or persons re-integrating with the community following incarceration. It is considered an intermediate step between emergency shelter and supportive housing and has its limits on how long a person or family can stay. - Shelter (e.g., a homeless shelter, emergency shelter, safe house for youth, or other short-term shelter): Short-term (30 days or less) facilities that provide sleeping arrangements and varying supports to people seeking emergency or urgent accommodation. - Hotel or motel - Hospital - Substance use or addictions treatment facility - Supervised consumption site (includes unregistered harm reduction sites) - Other healthcare facility (e.g., nursing home, long-term care facility, mental health unit, or other medical facility) - Emergency medical services (e.g., while in transit) - Workplace of the person who died - Correctional facility or police custody (e.g., federal correctional facility, a provincial/territorial correctional facility, young offender centre, remand centre, or police lock-up) - Public building (e.g., a shopping complex, marketplace, public bathroom, restaurant, bar, any recreational setting, gas station, airport, industrial setting, or other type of public building) - Outdoor public place (e.g., a park, bus stop, railroad track, public pool side, river, pond and lakeside, or other type of outdoor public place) - Public transportation |
| 6.6 | Took place in a vehicle | Indicates whether the person died in a vehicle directly outside of their place of death (e.g., the driveway of personal residence). |
| 6.7 | Took place outside | Indicates whether the person died directly outside their place of death (e.g., the driveway, porch, front walk, or backyard of their personal residence setting). |
| 6.8 | Estimated time of death | The approximate time when the death happened. Options include morning (6:00 to 11:59), noon or afternoon (12:00 to 17:59), evening (18:00 to 23:59), nighttime (00:00 to 5:59), or unknown or unobserved. |
| 6.9 | Date last known alive | The date when the person who died was last known to be alive. |
| 6.10 | Time last known alive | The estimated time that the person who died was last known to be alive. Options include morning (6:00 to 11:59), noon or afternoon (12:00 to 17:59), evening (18:00 to 23:59), nighttime (00:00 to 5:59), or unknown or unobserved. |
| 6.11 | Date the body was found | The date on which the body of the person who died was found. |
| 6.12 | Time the body was found | The approximate time when the body of the person who died was found. Options include morning (6:00 to 11:59), noon or afternoon (12:00 to 17:59), evening (18:00 to 23:59), nighttime (00:00 to 5:59), or unknown or unobserved. |
| 6.13 | Rigor mortis present when examined at scene | Indicates whether rigor mortis was present when the body was found. |
| 6.14 | Acute toxicity event location same as death location | Indicates if the acute toxicity event took place at the same location as the death. |
| 6.15 | Postal code of acute toxicity event | The postal code where the acute toxicity event precipitating death took place. |
| 6.16 | Place of acute toxicity event | The place where the acute toxicity event precipitating death took place. Options include:   - Personal residence setting (i.e., the home address of the person who died, including multi-tenant housing such as ‘rooming houses’) - Home of another person (a shared home or a friend or relative’s home) - Supportive or transitional housing (e.g., a group home or halfway home): Supportive housing is generally long-term accommodation that provides a varying range of supportive services depending on the needs of residents. It includes accommodation for people facing barriers to housing or living independently (for example, people with a mental illness, who use substances, or who have lost autonomy in older age). Transitional housing is intended to maximize independence and support the development of social, vocational, recreational, and life skills. These homes can be used to house children and youth, adults or seniors with chronic disabilities, persons seeking recovery from substance use, or persons re-integrating with the community following incarceration. It is considered an intermediate step between emergency shelter and supportive housing and has its limits on how long a person or family can stay. - Shelter (e.g., a homeless shelter, emergency shelter, safe house for youth, or other short-term shelter): Short-term (30 days or less) facilities that provide sleeping arrangements and varying supports to people seeking emergency or urgent accommodation. - Hotel or motel - Hospital - Substance use or addictions treatment facility - Supervised consumption site (includes unregistered harm reduction sites) - Other healthcare facility (e.g., nursing home, long-term care facility, mental health unit, or other medical facility) - Emergency medical services (e.g., while in transit) - Workplace of the person who died - Correctional facility or police custody (e.g., federal correctional facility, a provincial/territorial correctional facility, young offender centre, remand centre, or police lock-up) - Public building (e.g., a shopping complex, marketplace, public bathroom, restaurant, bar, any recreational setting, gas station, airport, industrial setting, or other type of public building) - Outdoor public place (e.g., a park, bus stop, railroad track, public pool side, river, pond and lakeside, or other type of outdoor public place) - Public transportation |
| 6.17 | Took place in a vehicle | Indicates whether the acute toxicity event took place in a vehicle directly outside of their place of death (e.g., the driveway of personal residence). |
| 6.18 | Took place outside | Indicates whether the acute toxicity event took place outside their place of death (e.g., the driveway, porch, front walk, or backyard of their personal residence setting). |
| 6.19 | Found in or near bed | Describes if the person who died was found in or near their bed, or in a location where there is information indicating a common sleeping arrangement (e.g., they were found on the couch, and there is mention in their C/ME file that this was where they usually slept at the time before their death). |
| 6.20 | Acute toxicity event took place on Indigenous reserve or land claim region | Indicates that the acute toxicity event took place on a First Nations reserve or a community governed by land-claim decisions or self-government agreements. |
| 6.21 | Date of acute toxicity event | The date on which the acute toxicity event precipitating death took place. |
| 6.22 | Weekday of the acute toxicity event | The weekday when the acute toxicity event precipitating death took place. |
| 6.23 | Time of the acute toxicity event | The approximate time when the acute toxicity event precipitating death took place. This may be the approximate time when the witness observed the person who died lose consciousness, fall asleep, or present other symptoms of an acute toxicity event. Options include morning (6:00 to 11:59), noon or afternoon (12:00 to 17:59), evening (18:00 to 23:59), nighttime (00:00 to 5:59), or unknown or unobserved. |
| 6.24 | Presence of drugs or alcohol at the scene | Indicates if drugs or alcohol were present at the scene of death. The scene refers to the place where the body was found or the acute toxicity event took place and can include any area that contains evidence related to the acute toxicity event itself. Options include (multiple selections allowed) no alcohol or drug were present; illicit drugs were present, prescription drugs were present, alcohol was present, or unknown. |
| 6.25 | Apparent mode of drug use | Describes the apparent mode of drug consumption or use as per the body and scene examination. Options include (multiple selections allowed) likely injection, likely oral, likely nasal insufflation or intranasal (snorting), likely smoking, likely transdermal (patches), other, or unknown. |
| 6.26 | Evidence for apparent mode of drug use | Describes how the apparent mode of drug use was determined (i.e., oral may have been selected based on the presence of intact pills in the stomach discovered during autopsy). |
| 6.27 | Drug paraphernalia at scene | Refers to any equipment that was used to produce, conceal, and consume drugs found at the scene. Options include (multiple selections allowed) nothing was there; syringe, tourniquet, or evidence of injection; pipes; tin foil or spoon with drug residue; hollow tube, straw, chop lines, rolled-up papers, or evidence of snorting; marijuana or evidence of marijuana (e.g., bong); drug baggies; lighter, razors, or cutting tools; residue on smooth surface (mirror, table, etc.); needle exchange kit contents; pill bottle, medications, dispill, or medication packaging system; powdery substance; glow stick, surgical/dust; pacifiers and lollipops; other; or unknown. |
| 6.28 | Using drugs in presence of others | Indicates if substances were taken in the presence of others prior to the fatal acute toxicity event. Options include no, yes, alcohol only, or unknown. |
| 6.29 | Evidence that acute toxicity event was witnessed | Describes if the fatal acute toxicity event was witnessed by one or more individuals (e.g., family, friends, acquaintance, passers-by, etc.). Options include:   - Yes, person was alive when found, and showing symptoms - No, person was deceased when found and there was no evidence that the acute toxicity event was witnessed - Unclear, person was unconscious or unresponsive when found, it was unknown if they had already died - Unclear, individual was thought to be asleep - Unknown |
| 6.30 | Witness observations | If the acute toxicity event may have been witnessed, describes witness observations of any signs or symptoms displayed by the person who died (whether they knew in the moment an acute toxicity event had occurred or not). Options include (multiple selections allowed) snoring or gurgling sound, seizure, difficulty breathing, vomiting, loss of balance, confusion, pinpoint pupils, dilated pupils, sudden aggression, sweat, weak or rapid pulse, unconscious or unresponsive, asleep, blue lips or fingernails, foaming at mouth, other, or unknown. |
| 6.31 | Witness believed situation to be an acute toxicity event | If the acute toxicity event may have been witnessed, indicates if the witness believed the situation to be an acute toxicity (overdose) event at the time of the fatal event. Options include yes; knew the person was intoxicated but did not consider it an acute toxicity event (i.e., a serious situation); did not know person was intoxicated; or unknown. |
| 6.32 | Prompt action taken by witness | If the acute toxicity event may have been witnessed, describes the action(s) taken by the individuals present near or at the scene in seeking medical support in the moments when the acute toxicity event occurred. If multiple encounters with the case took place, captures actions taken during the first encounter. Options include (multiple selections allowed) no action taken, resuscitative activities (unspecified), took individual to a medical facility, called 911, called someone else, CPR attempted, rescue breathing administered, stimulation administered, epinephrine administered, oxygen provided, other, or unknown. |
| 6.33 | Reason why no action was taken | In situations where no action was taken by a witness, describes the reasons for this given by the witness. |
| 6.34 | Subsequent action taken by witness | If the acute toxicity event may have been witnessed, describes the action(s) taken by the individuals present near or at the scene in seeking medical support in the moments when the acute toxicity event occurred. If multiple encounters with the case took place, captures actions taken during the any subsequent encounters. Options include (multiple selections allowed) no action taken, resuscitative activities (unspecified), took individual to a medical facility, called 911, called someone else, CPR attempted, rescue breathing administered, stimulation administered, epinephrine administered, oxygen provided, other, or unknown. |
| 6.35 | Multiple fatality event | Describes if the fatal incident claimed the lives of more than one person. |
| 6.36 | First aid provided by first responders | Describes first aid interventions provided by the first responders (including emergency medical services, police, and fire fighters) once they arrived on the scene. Options include (multiple selections allowed) no first responders attended the scene, no intervention, resuscitative activities (unspecified), CPR given, rescue breathing administered, stimulation administered, epinephrine administered, provided oxygen, other, or unknown. |
| 6.37 | First aid provided at a health care setting | Describes the interventions provided by staff once the person was transferred to a healthcare setting. Options include (multiple selections allowed) person was not transferred to a health care setting, no intervention, resuscitative activities (unspecified), CPR given, rescue breathing administered, stimulation administered, epinephrine administered, oxygen provided, admitted to a health care setting (e.g., the intensive care unit), other, or unknown. |
| 6.38 | Who attended the scene | Indicates the specific first responders or care providers present at the scene. Options include emergency medical services, fire services, law enforcement, or hospital staff. |
| 6.39 | Was naloxone administered? | Indicates whether the person who died received naloxone over the course of the response to the acute toxicity event. |
| 6.40 | Indicate who administered naloxone | Describes the individual(s) who administered naloxone in response to the acute toxicity event. Options include (multiple selections allowed) emergency medical services, fire services, law enforcement, hospital staff, bystanders, other, or unknown. |
| 6.41 | Number of doses administered | Indicates the number of doses of naloxone that were administered to the person who died. |
| 6.42 | Evidence of possible medication error | Describes whether the C/ME report provides evidence that the person who died had mistakenly consumed the medication (e.g., methadone in a juice bottle left in the fridge) or there was a medical error in what they were supposed to consume. This refers to the drug obtained before death that contributed to the death. Options include possible hospital medication error; possible community pharmacy medication error; drug apparently unknowingly consumed; other; or unknown or no evidence of medication error. |
| 6.43 | Biological substance | Indicates if there is circumstantial evidence that biological substances caused or contributed to the death. Options include insulin; a substance other than insulin; or unknown or no evidence of involvement of biological substances. |
| 6.44 | Death certificate available | Indicates whether the death certificate was attached to the C/ME file of the person who died. |
| 6.45 | Manner of death | The manner of death reflects the circumstances surrounding the death [10], as determined by the C/MEs who collected and examined information on how and why the death occurred. In most jurisdictions, there are only five such categories: natural, accident, suicide, homicide, and undetermined. This study only included cases with the following manners of death:   - Accidental: A death that is caused by an injury and there is no obvious intent to cause death [11]. In this study, an accidental manner of death indicates an unintentional acute toxicity. - Suicide: When someone dies and the evidence indicates that the person intended to cause their own death [11]. In this study, suicide as the manner of death indicates an intentional acute toxicity. - Undetermined: When a complete death investigation does not provide enough evidence to determine the manner of death [11]. In this study, an undetermined manner indicates the manner of death could not be assigned based on the available evidence. |
| 6.46 | Immediate cause of death | The immediate cause of death as specified in the death certificate, autopsy summary (or post-mortem), or C/ME report. The cause of death is defined as the disease or injury that set into motion a chain of events that ended in death (with no implication of any time limit). |
| 6.47 | Time interval between onset and death for the immediate cause of death | The time interval between onset and death for the immediate cause of death, copied exactly as it appears in the death certificate. |
| 6.48 | Death certificate available | Indicates whether the death certificate was attached to the C/ME file of the person who died. |
| 6.49 | Antecedent cause of death A, B, C, and D | The conditions giving rise to the immediate cause of death, stating the underlying cause last, as assessed by the pathologist after completing the postmortem report. These are copied exactly as they appear in the death certificate. |
| 6.50 | Time interval between onset and death for antecedent causes A, B, C, and D | The time interval between onset and death for antecedent causes A, B, C, and D, copied exactly as they appear in the death certificate. |
| 6.51 | Significant conditions 1, 2, and 3 | The three significant conditions listed in the death certificate and copied exactly as they appear. |
| 6.52 | Recommendations | A verbatim copy of any recommendations made by the C/ME in the C/ME report. |

## Toxicology findings

Table 7. Toxicology variables collected for our national chart review study of substance-related acute toxicity deaths.

| No. | Variable name | Description |
| --- | --- | --- |
| 7.1 | Toxicology report available | Indicates if a toxicology report is available for the C/ME case file and the date of the report. |
| 7.2 | Post-mortem examination completed | Indicates if a post-mortem examination was conducted for the person who died. |
| 7.3 | Reason for unavailability of toxicology report | If a toxicology investigation was not done, indicate the reason for the unavailability of a sample for performing toxicological analyses. Options include decomposition, admission blood not being available, a prolonged hospital stay, other, or unknown. |
| 7.4 | Rapid toxicology report available | Indicates if a rapid toxicology report or test result was available for data abstraction (e.g., testing performed by a hospital laboratory). |
| 7.5 | Cause of death only described as multi-drug toxicity or combined toxicity with no substances specified | Indicates if the C/ME file only described the cause of death as ‘multi-drug toxicity’ or ‘combined toxicity’ without listing the specific substances that caused or contributed to the death. |
| 7.6 | No evidence in the C/ME file to suggest a pharmaceutical origin for any detected substance | Indicates if there is no evidence anywhere in the C/ME file to suggest a pharmaceutical origin for any of the substances detected on toxicology. Evidence of a pharmaceutical origin may include:   - Record of prescription information (identified during investigation interviews with health care providers, in medical files, or systematically linked to a prescription database) - Scene evidence of a prescription drug prescribed to the person who died (e.g., a prescription bottle with their name on the label) - Scene evidence of a prescription drug not prescribed to the person who died (e.g., a prescription bottle with someone else's name on it) - Other information identified during the investigation (e.g., report by family or witness) |
| 7.7 | **Opioids** detected or that contributed to death according to the C/ME file, as well as evidence regarding their source and origin. This includes 6-hydroxy-oxymorphone, 6-monoacetylmorphine (6-MAM), acetylfentanyl, buprenorphine [norbuprenorphine], butyrylfentanyl [carboxybutyrfentanyl, hydroxybutyrfentanyl], carfentanil, codeine, cyclopropyl/crotonyl fentanyl, despropionyl-fentanyl (4-ANPP), dextrorphan/levorphanol, diacetylmorphine (heroin), furanylfentanyl, fentanyl, hydrocodone [norhydrocodone], hydromorphone, hydromorphone-3-glucoronide, meperidine [normeperidine], methadone [2-ethylidene-1,5-dimethyl-3,3-diphenylpyrrolidine (EDDP), 2-ethyl-5-methyl-3,3-diphenylpyrroline], morphine, morphine-3-glucuronide, morphine-6-glucuronide,norcodeine, norfentanyl, normorphine, oxycodone [noroxycodone], oxymorphone, oxymorphone-3-glucoronide, propoxyphene/dextropropoxyphene [norpropoxyphene], tapentadol, tramadol [N-desmethyltramadol (nortramadol), O-desmethyltramadol], U-47700, and other opioids.  **Antipsychotic** substances detected or that contributed to death according to the C/ME file, as well as evidence regarding their source and origin. This includes aripiprazole, asenapine, clozapine [desmethylclozapine (norclozapine)], fluphenazine, haloperidol, lurasidone, olanzapine, paliperidone (9-hydroxyrisperidone), quetiapine [N-desalkylquetiapine (norquetiapine)], risperidone, ziprasidone, and other antipsychotic substances.  **Benzodiazepines** detected or that contributed to death according to the C/ME file, as well as evidence regarding their source and origin. This includes alprazolam [α-hydroxyalprazolam], bromazepam [hydroxybromazepam], clobazam [N-desmethylclobazam (norclobazam), 4-hydroxyclobazam], clonazepam [7-aminoclonazepam], diazepam, etizolam [α-hydroxyetizolam], flurazepam [hydroxyethyl-flurazepam], lorazepam, lorazepam-glucuronide, N-desalkylflurazepam (norflurazepam), nitrazepam [7-aminonitrazepam], nordiazepam, oxazepam, temazepam, and other benzodiazepines.  **Antidepressants** detected or that contributed to death according to the C/ME file, as well as evidence regarding their source and origin. This includes amitriptyline, buproprion [hydroxybuproprion, threohydroxybupropion, erythrohydroxybupropion], citalopram/escitalopram [desmethylcitalopram, didesmethylcitalopram], clomipramine [desmethylclomipramine], desipramine, desvenlafaxine [O-desmethylvenlafaxine], doxepin [N-desmethyldoxepin (nordoxepin)], duloxetine, fluoxetine [norfluoxetine], fluvoxamine, imipramine, m-chlorophenylpiperazine (mCPP), mirtazapine [N-desmethylmirtazapine, mirtazapine-N-oxide], nortriptyline, paroxetine, sertraline [N-desmethylsertraline (norsertraline)], trazodone, venlafaxine, and other antidepressants.  **Stimulants and hallucinogens** detected or that contributed to death according to the C/ME file, as well as evidence regarding their source and origin. This includes 4-hydroxyamphetamine, 4-hydroxymethamphetamine, amphetamine, caffeine, cocaine (crack) [benzoylecogonine, anhydroecgonine methyl ester, ecgonine methyl ester], cocaethylene, dextroamphetamine, ethylphenidate, ketamine [norketamine, hydroxynorketamine, hydroxyketamine], lysergic acid diethylamide (LSD) [2-oxo-3-hydroxy-LSD], methamphetamine [N-methylamphetamine], 3,4-methylenedioxyamphetamine (MDA), methylenedioxymethamphetamine (MDMA, ecstasy), methylphenidate, paramethoxyamphetamine (PMA), paramethoxymethamphetamine (PMMA), phencyclidine (PCP), psilocybin [psilocyn], ritalinic acid, tetrahydrocannabinol (THC) [tetrahydrocannabinol carboxylic acid (THC-COOH)], and other stimulants and hallucinogens.  **Other substances** detected or that contributed to death according to the C/ME file, as well as evidence regarding their source and origin. Variables include ethanol (alcohol) [acetaldehyde, ethyl glucuronide, ethyl sulfate], acetaminophen, acetone, aminochloropyridine (ACP), baclofen, butane, clonidine [p-hydroxyclonidine], cyclobenzaprine [N-desmethylcyclobenzaprine (norcyclobenzaprine)], dextromethorphan, diltiazem, diphenhydramine, gabapentin, gammahydroxybutyrate (GHB), isopropanol (isopropyl alcohol), levamisole, loperamide, methanol [formaldehyde, formate], nabilone, naloxone, orphenadrine, phenobarbital, phenylethylmalonamide (PEMA), pregabalin, primidone, pseudoephedrine/ephedrine, selegiline [desmethylselegiline], xylazine, zolpidem, zopiclone, and other substances. | Describes opioids, antipsychotics, benzodiazepines, antidepressants, stimulants, hallucinogens, other drugs, and alcohol that were tested for and detected during toxicological analyses. It also confirms if the substance was described as contributing to death and refers to the possible source and origin from which the substance was obtained. Variables include:   - Detected: The substance was tested for and identified in the blood, urine, or any related specimen from the person who died. This may be a quantitative or qualitative value (for example, 'detected' or 'traces'). - Drug contributed to death: The substance was identified as contributing to the death according to the C/ME report, autopsy summary, or the death certificate. Indicates that the drug concentration was sufficiently high to cause death, or a combination of drugs including this drug was present at a clinically significant concentration resulting in death. This may have been affected by personal factors (e.g., tolerance), proximal factors (e.g., mode of use), and environmental factors (e.g., temperature). - Evidence of pharmaceutical origin, source unknown: Evidence that the substance was produced by a pharmaceutical company but unknown whether prescribed to case or diverted. - Evidence of prescribed pharmaceutical source: Evidence that substance was produced by a pharmaceutical company and prescribed to the case. - Evidence of diverted pharmaceutical source: Evidence that substance was produced by a pharmaceutical company and not prescribed to the case.   Evidence of a pharmaceutical origin may include:   - Record of prescription information (identified during investigation interviews with health care providers, in medical files, or systematically linked to a prescription database) - Scene evidence of a prescription drug prescribed to the person who died (e.g., a prescription bottle with their name on the label) - Scene evidence of a prescription drug not prescribed to the person who died (e.g., a prescription bottle with someone else's name on it) - Other information identified during the investigation (e.g., report by family or witness) |
| 7.8 | Blood alcohol content | Indicates the blood alcohol content as measured on the toxicology report. |
| 7.9 | Detected in vitreous fluid | Indicates whether ethanol was detected in a vitreous fluid specimen. |
| 7.10 | Potential relationship to putrefaction | Indicates whether the toxicology report or post-mortem examination provides evidence to suggest that the formation of ethanol (alcohol), isopropanol (isopropyl alcohol), or methanol was a result of putrefaction/decomposition. |

## References

1. Statistics Canada. Dictionary, Census of Population, 2016, Census subdivision (CSD). November 16, 2016. <https://www12.statcan.gc.ca/census-recensement/2016/ref/dict/geo012-eng.cfm> [accessed Feb 1, 2019].
2. Statistics Canada. 2021. Postal Code Conversion File Plus (PCCF+). March 6, 2017. <https://www150.statcan.gc.ca/n1/en/catalogue/82F0086X> [accessed Jul 18, 2022].
3. Subedi R, Roshanafshar S, Greenberg TL. 2020. Developing meaningful categories for distinguishing levels of remoteness in Canada (Catalogue No. 11-633-X-026). August 11, 2020. <https://www150.statcan.gc.ca/n1/pub/11-633-x/11-633-x2020002-eng.htm> [accessed Jul 18, 2022].
4. Statistics Canada. 2019. The Canadian Index of Multiple Deprivation. June 12, 2019. <https://www150.statcan.gc.ca/n1/en/catalogue/45200001> [accessed Jul 18, 2022].
5. Government of Ontario. 2018. Data standards for the identification and monitoring of systemic racism. April 13, 2022. <https://www.ontario.ca/document/data-standards-identification-and-monitoring-systemic-racism> [accessed Feb 1, 2019].
6. Gaetz S BC, Friesen A, Harris B, Hill C, Kovacs-Burns K, Pauly B, Pearce B, Turner A, Marsolais A. 2012. Canadian definition of homelessness. <https://www.homelesshub.ca/sites/default/files/COHhomelessdefinition.pdf> [accessed Jul 18, 2022].
7. Statistics Canada. 2016. National Occupational Classification (NOC) 2016 Version 1.2. <https://www23.statcan.gc.ca/imdb/p3VD.pl?Function=getVD&TVD=1204813&CVD=1204814&CLV=0&MLV=4&D=1> [accessed Feb 1, 2019].
8. Statistics Canada. 2016. North American Industry Classification System (NAICS) Canada 2017 Version 3.0. 2017. <https://www23.statcan.gc.ca/imdb/p3VD.pl?Function=getVD&TVD=1181553> [accessed Feb 1, 2019].
9. Substance Abuse and Mental Health Services Administration’s Trauma and Justice Strategic Initiative. 2014. SAMHSA’s concept of trauma and guidance for a trauma-informed approach. July 2014. <https://ncsacw.acf.hhs.gov/userfiles/files/SAMHSA_Trauma.pdf> [accessed Feb 1, 2019].
10. Government of Nova Scotia. 2021. Nova Scotia Medical Examiner Service. <https://novascotia.ca/just/cme/> [accessed Feb 1, 2019].
11. Government of Alberta. 2022. Death investigation process. <https://www.alberta.ca/death-investigation-process.aspx> [accessed Feb 1, 2019].
