# Supplementary material for: Substance-Related Acute Toxicity Deaths in Canada From 2016 to 2017: Protocol for a Retrospective Chart Review Study of Coroner and Medical Examiner Files
Source: JMIR Public Health Surveill. 2025 Apr 10;11:e49981. doi: 10.2196/49981 (PMC12022517; doi:10.2196/49981)
Supplement: Multimedia Appendix 2 [file publichealth_v11i1e49981_app2.docx]

Substance-related acute toxicity deaths in Canada from 2016 to 2017: A protocol for a retrospective chart review study of coroner and medical examiner files

# Appendix 2: Screenshots of the study’s data collection tool.

## Case definition

Figure 1. Screenshot of the case definition tab of the data collection tool for our national chart review study of substance-related acute toxicity deaths. Red variables were only collected at the request of the chief coroner or medical examiner’s office and not shared with the national study team.


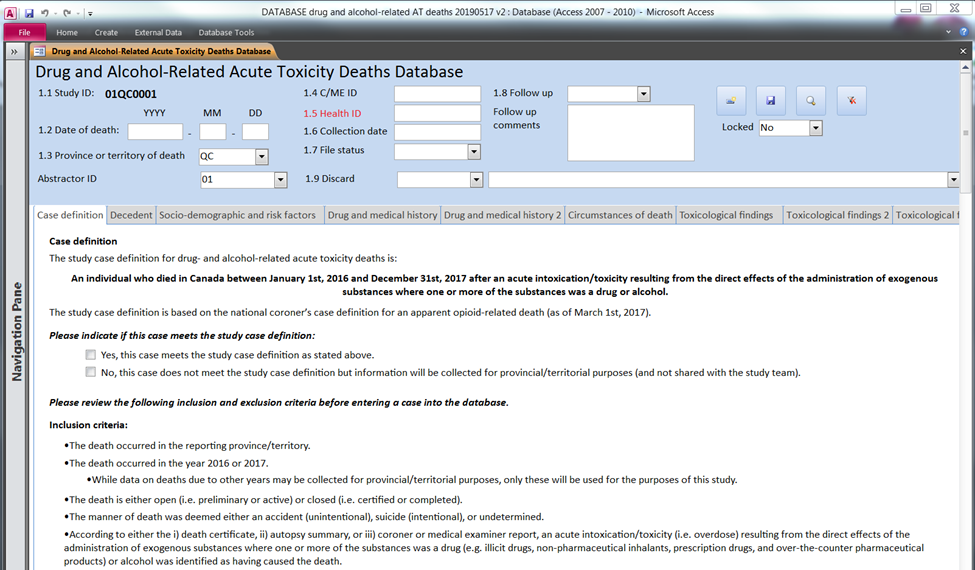


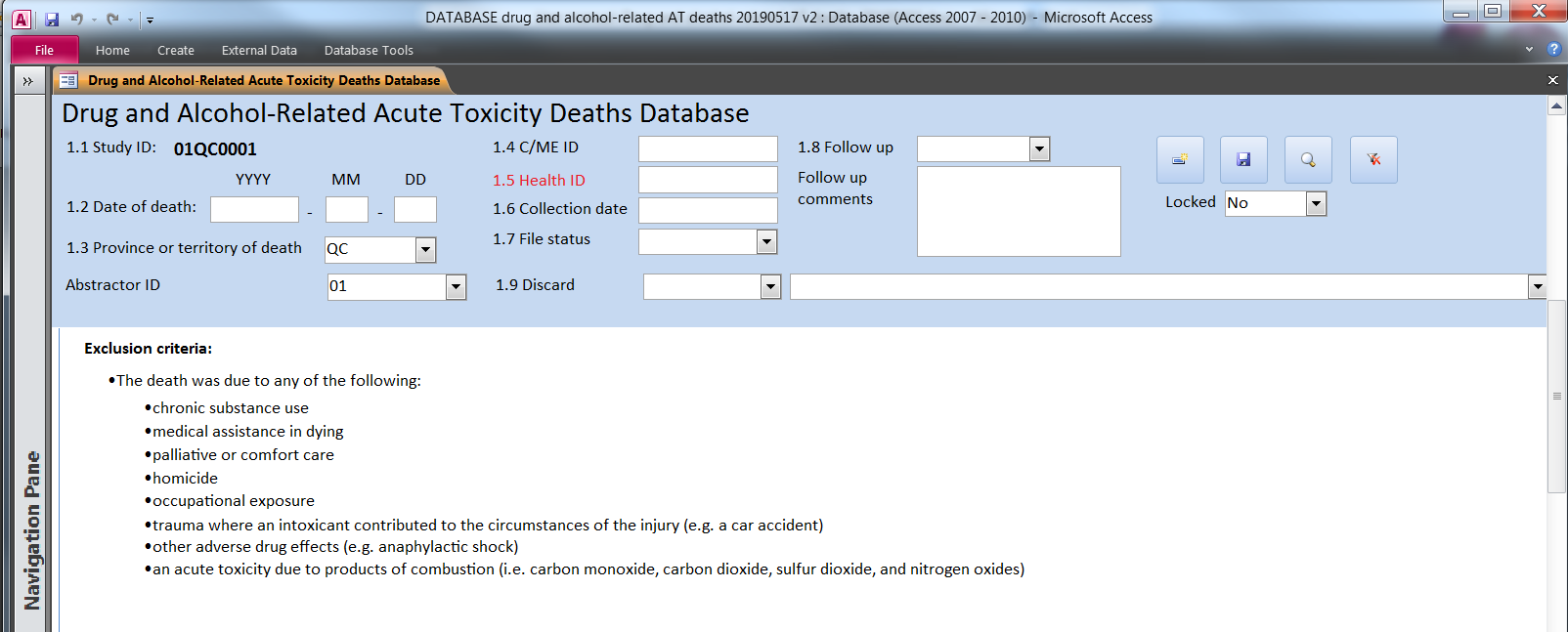


## Decedent information

Figure 2. Screenshot of the decedent information tab of the data collection tool for our national chart review study of substance-related acute toxicity deaths. Red variables were only collected at the request of the chief coroner or medical examiner’s office and not shared with the national study team.


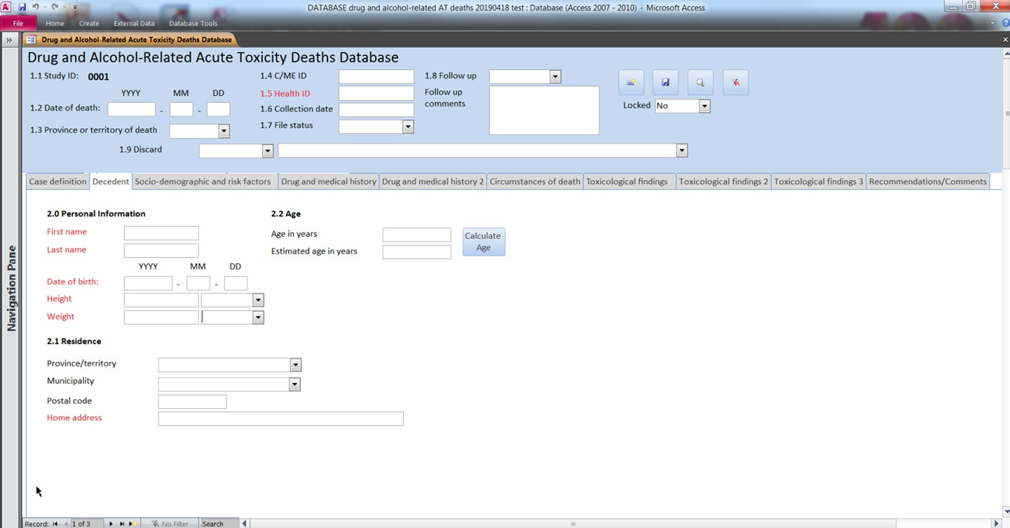


## Demographic and socioeconomic factors

Figure 3. Screenshot of the sociodemographic and risk factors tab of the data collection tool for our national chart review study of substance-related acute toxicity deaths. Red variables were only collected at the request of the chief coroner or medical examiner’s office and not shared with the national study team.


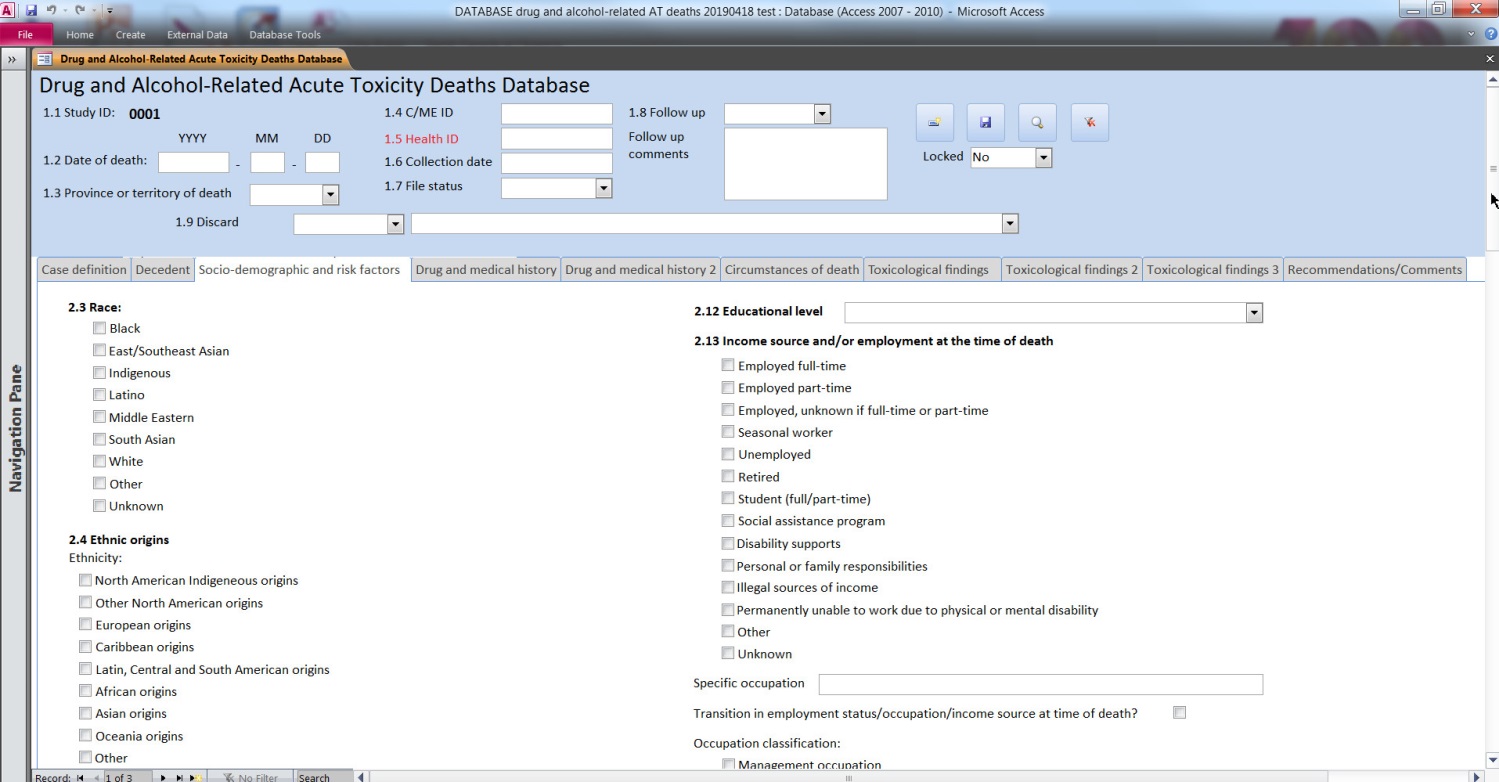


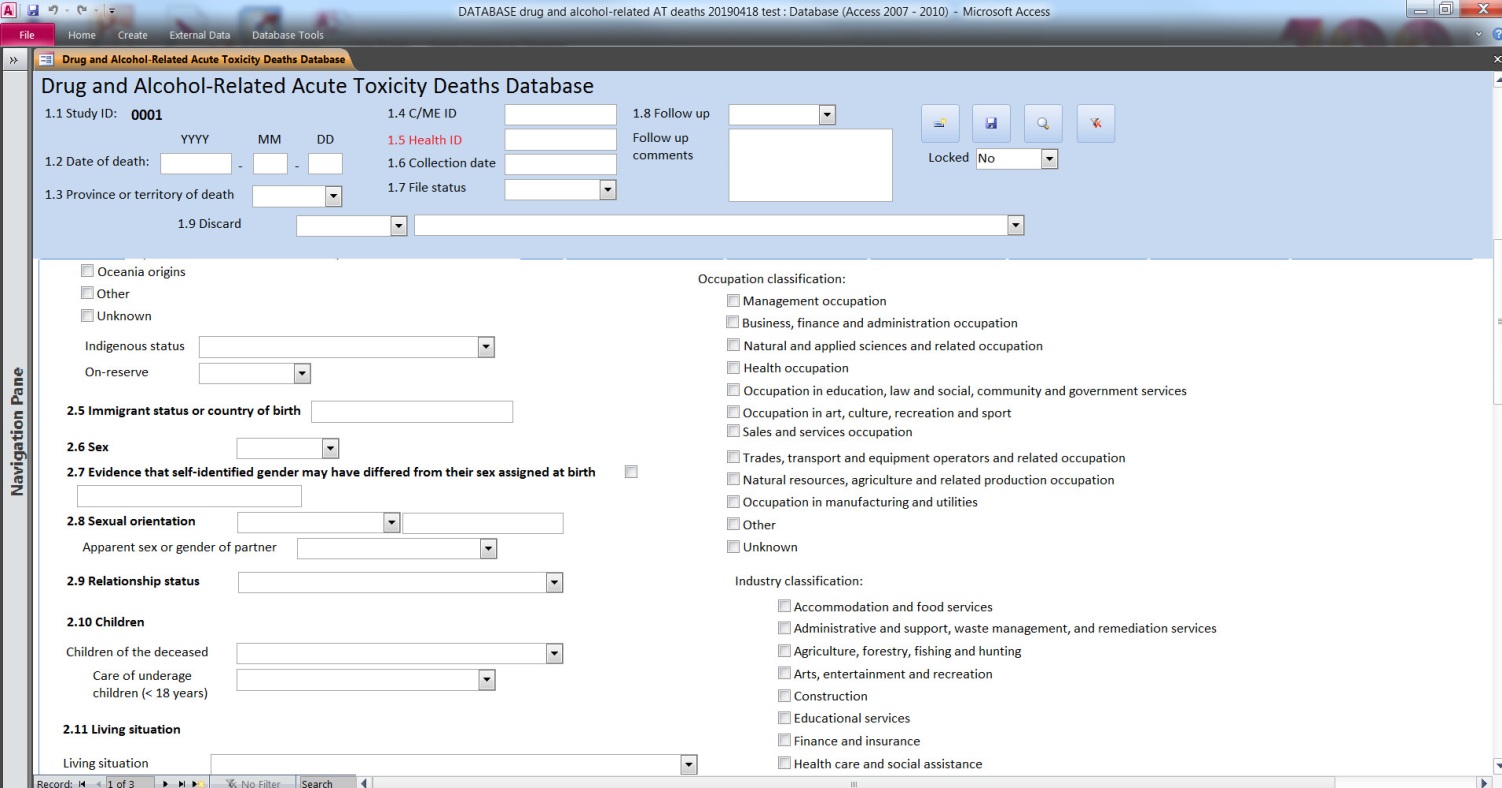


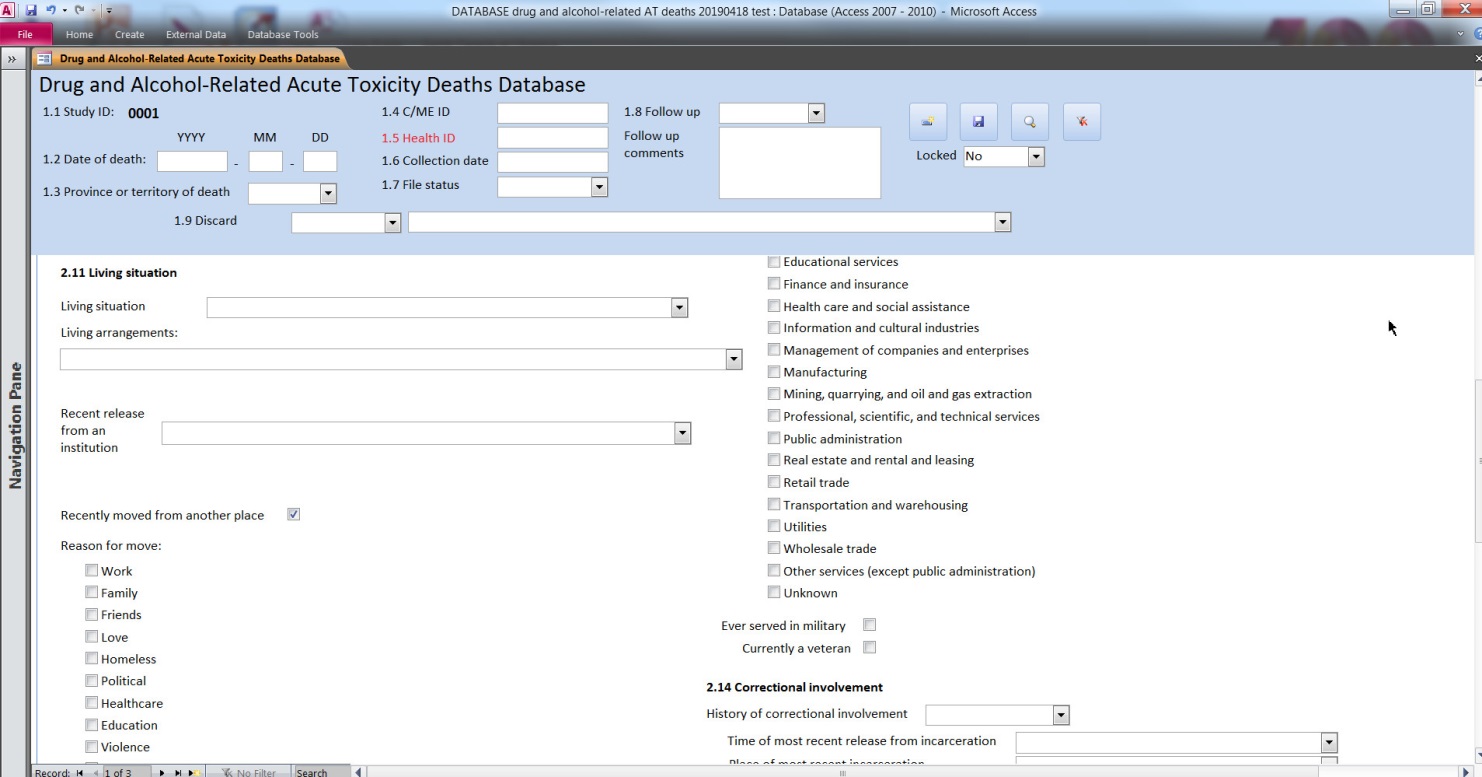


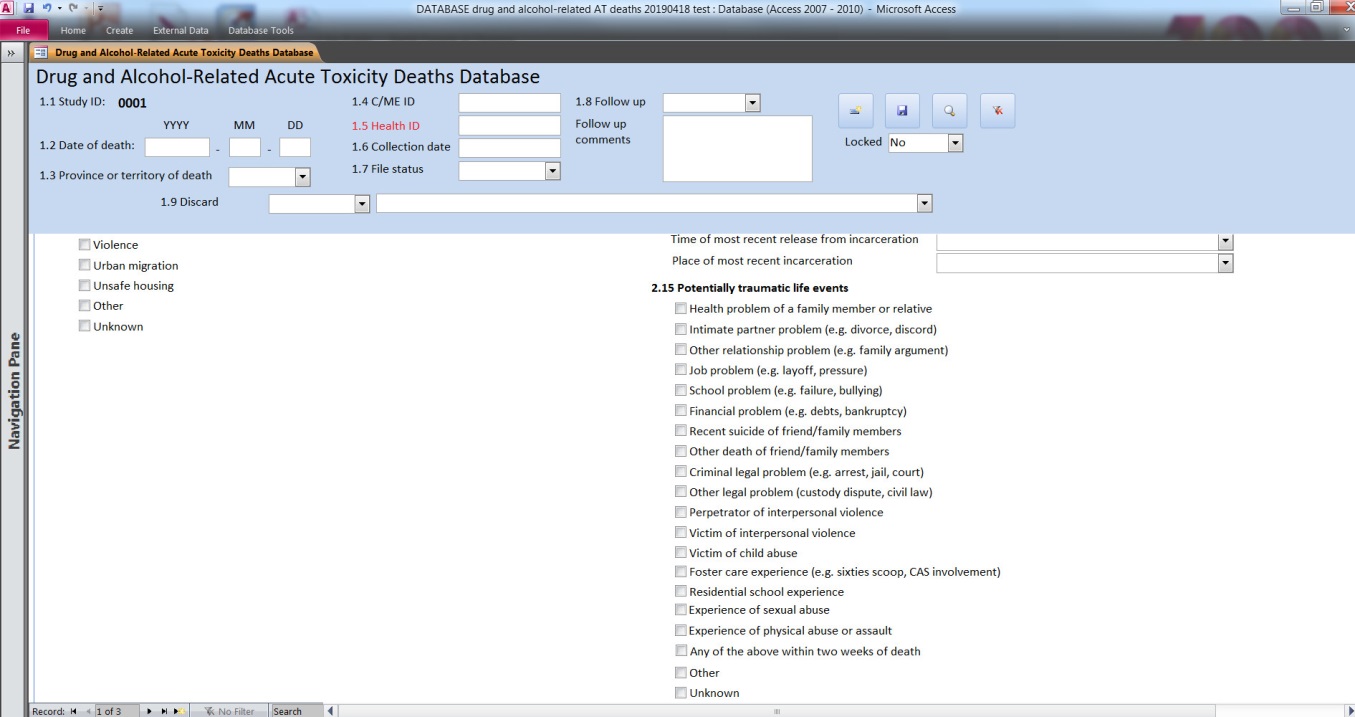


## Drug and medical history

Figure 4. Screenshot of the drug and medical history tab of the data collection tool for our national chart review study of substance-related acute toxicity deaths. Red variables were only collected at the request of the chief coroner or medical examiner’s office and not shared with the national study team.


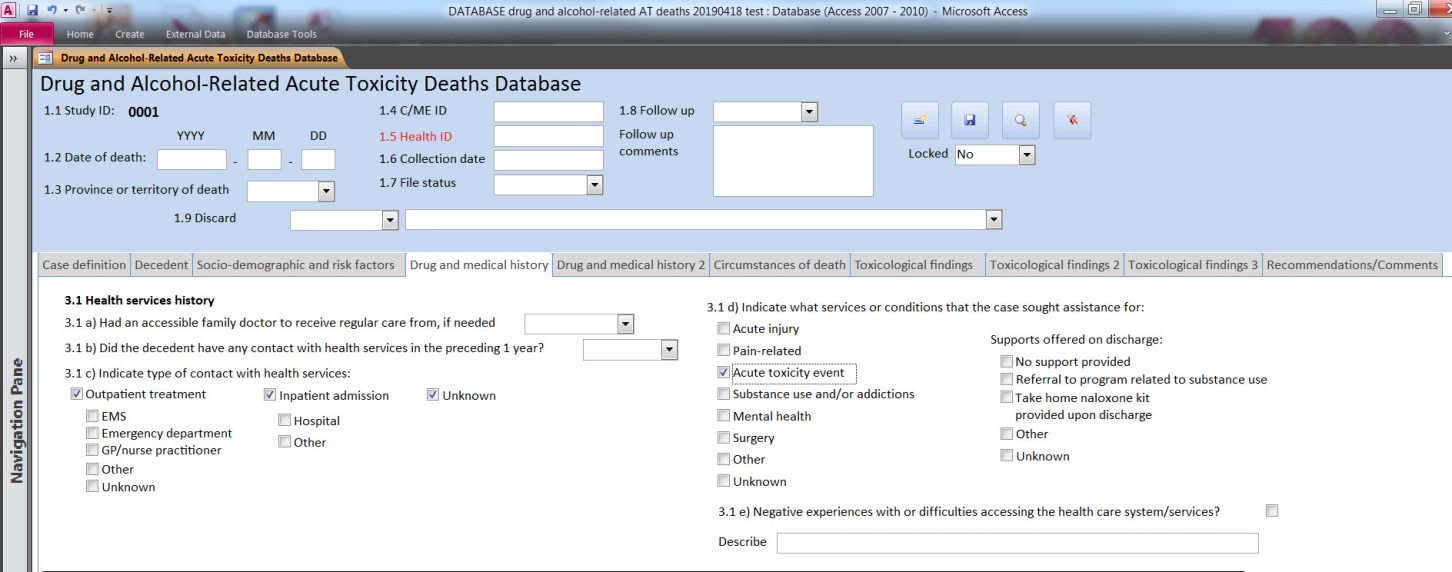


**
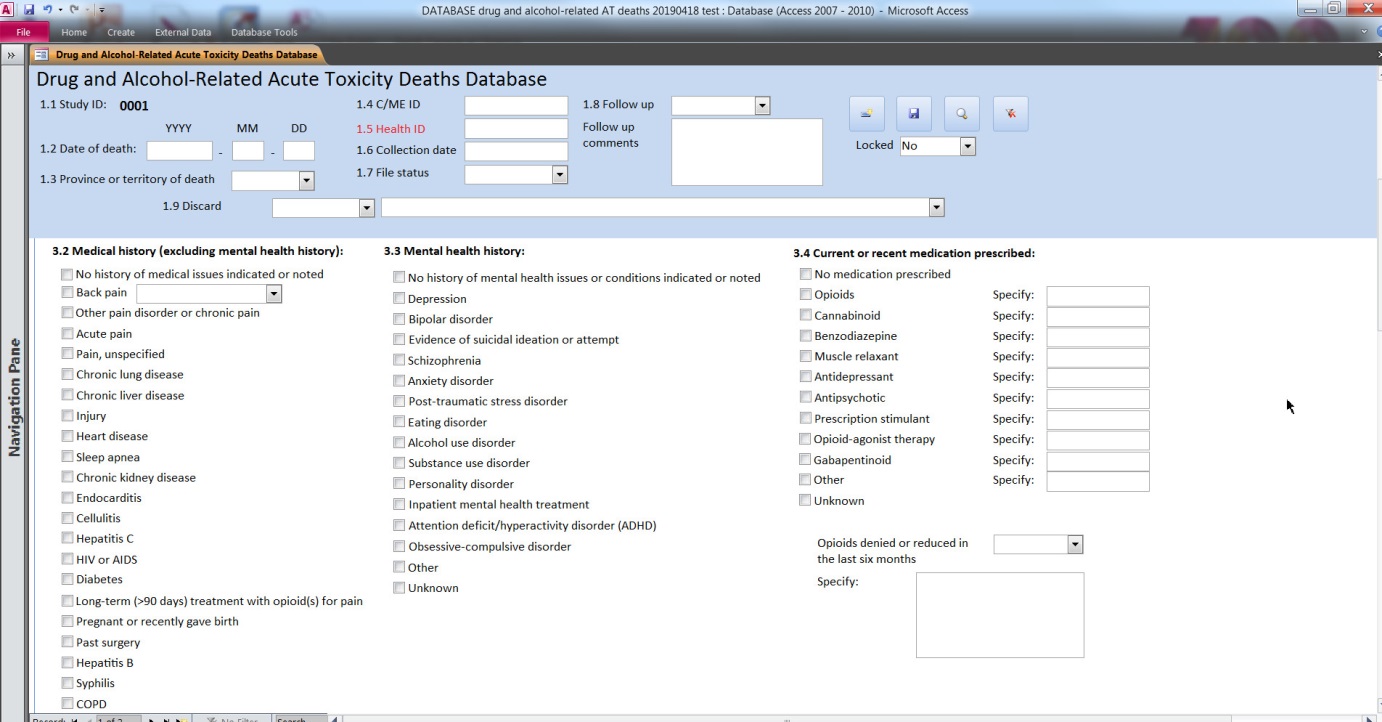
**


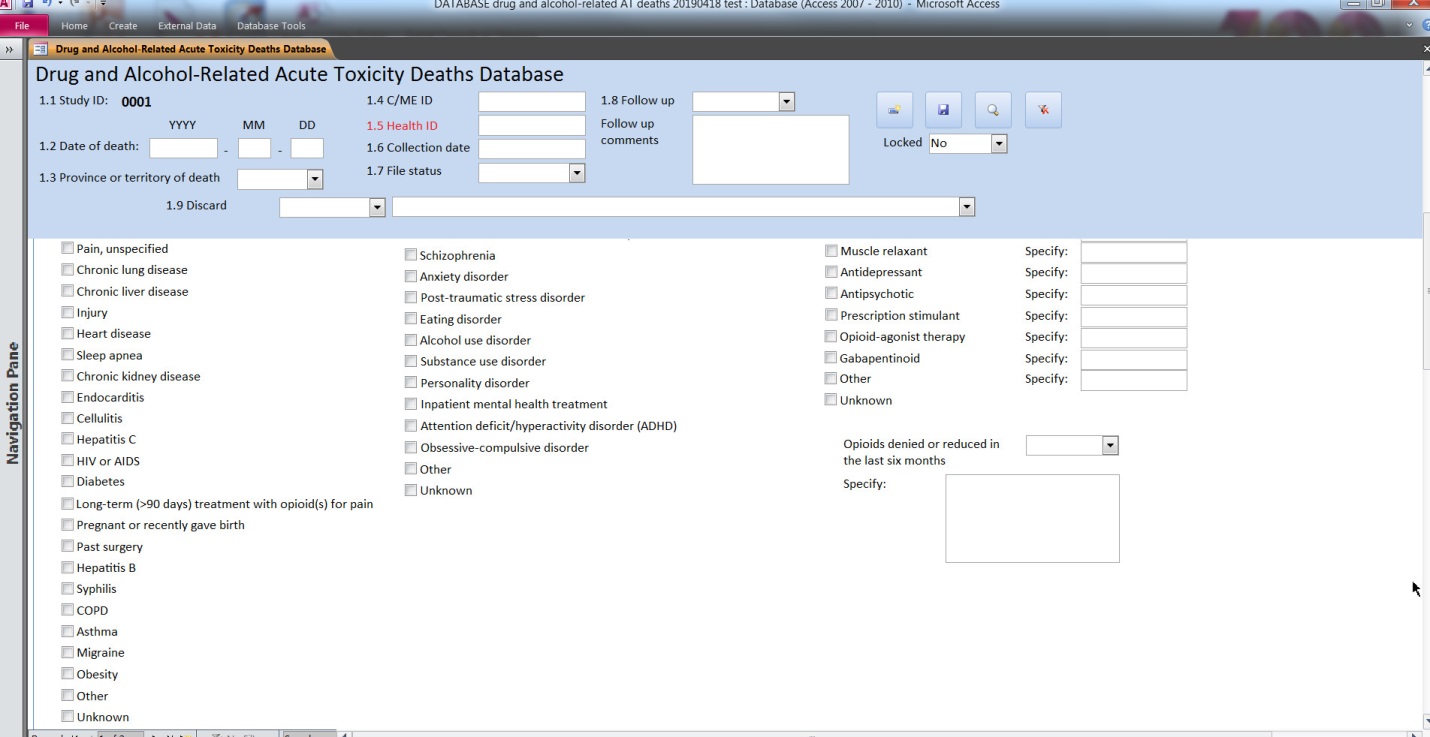


## Substance use history

Figure 5. Screenshot of the drug and medical history 2 tab (describing substance use history) of the data collection tool for our national chart review study of substance-related acute toxicity deaths. Red variables were only collected at the request of the chief coroner or medical examiner’s office and not shared with the national study team.


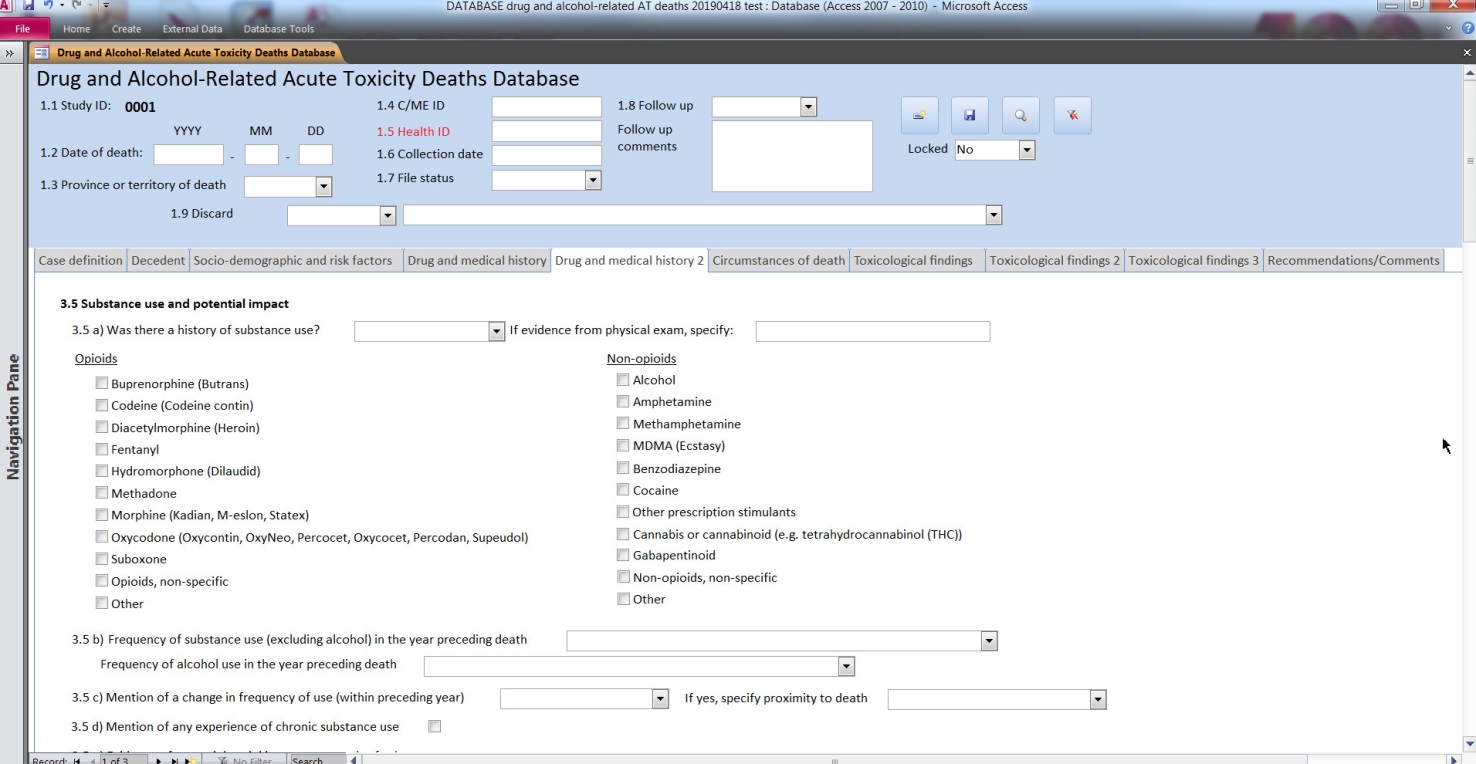


**
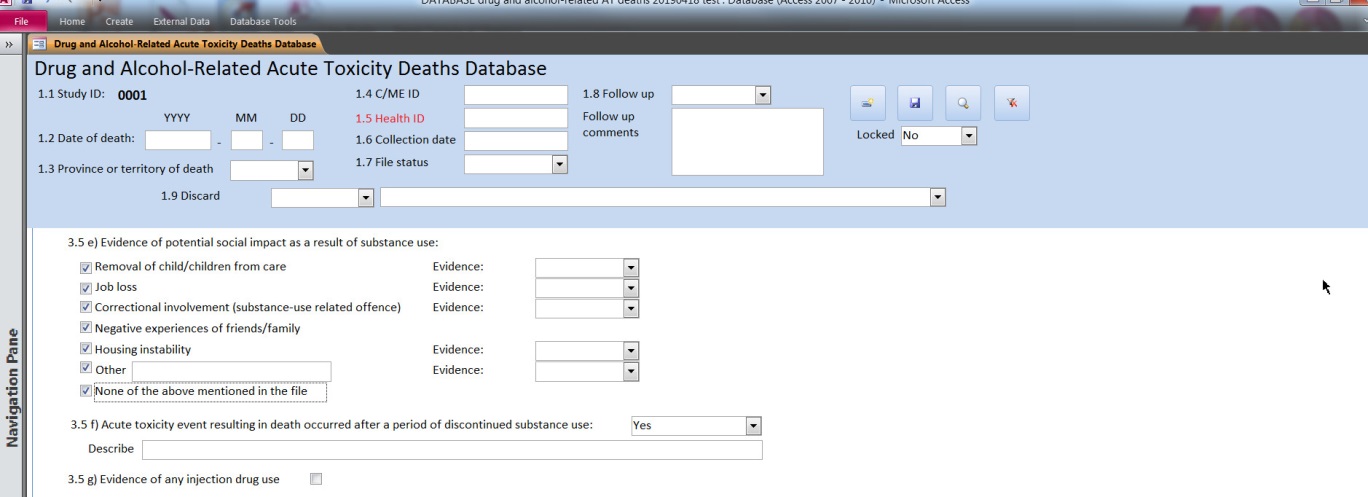
**


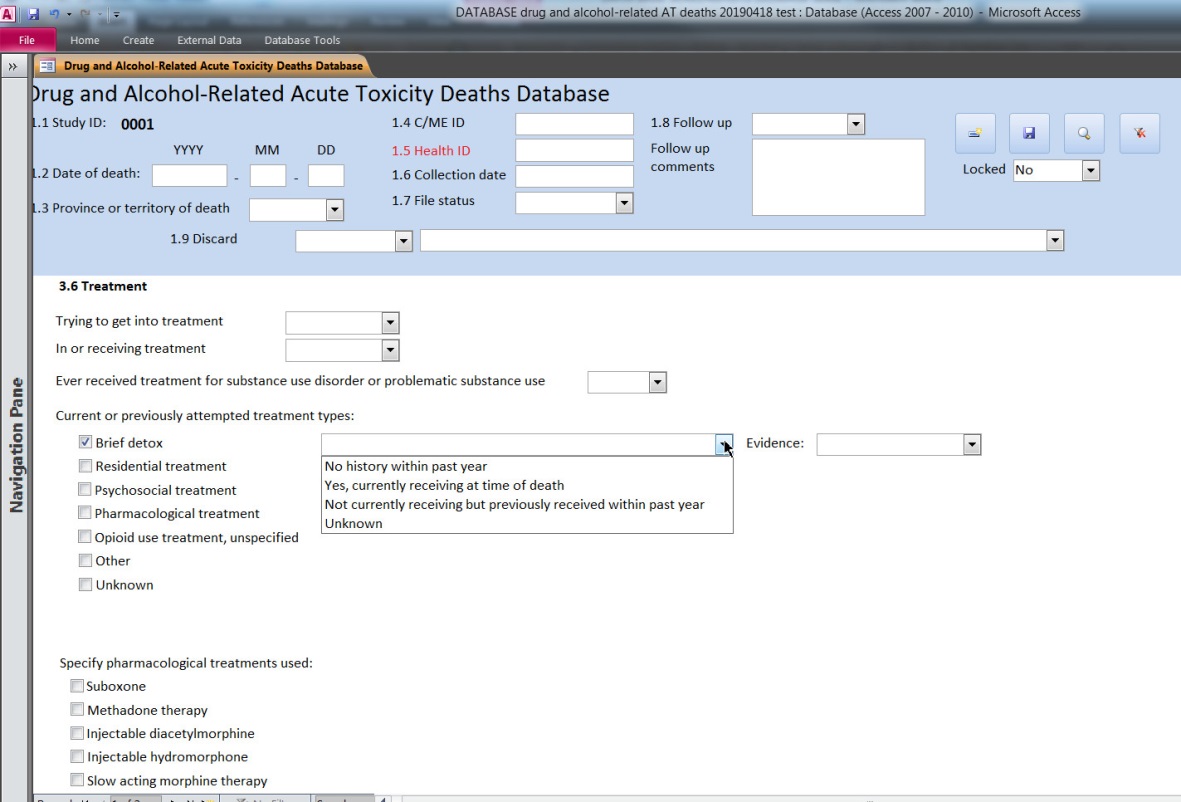


**
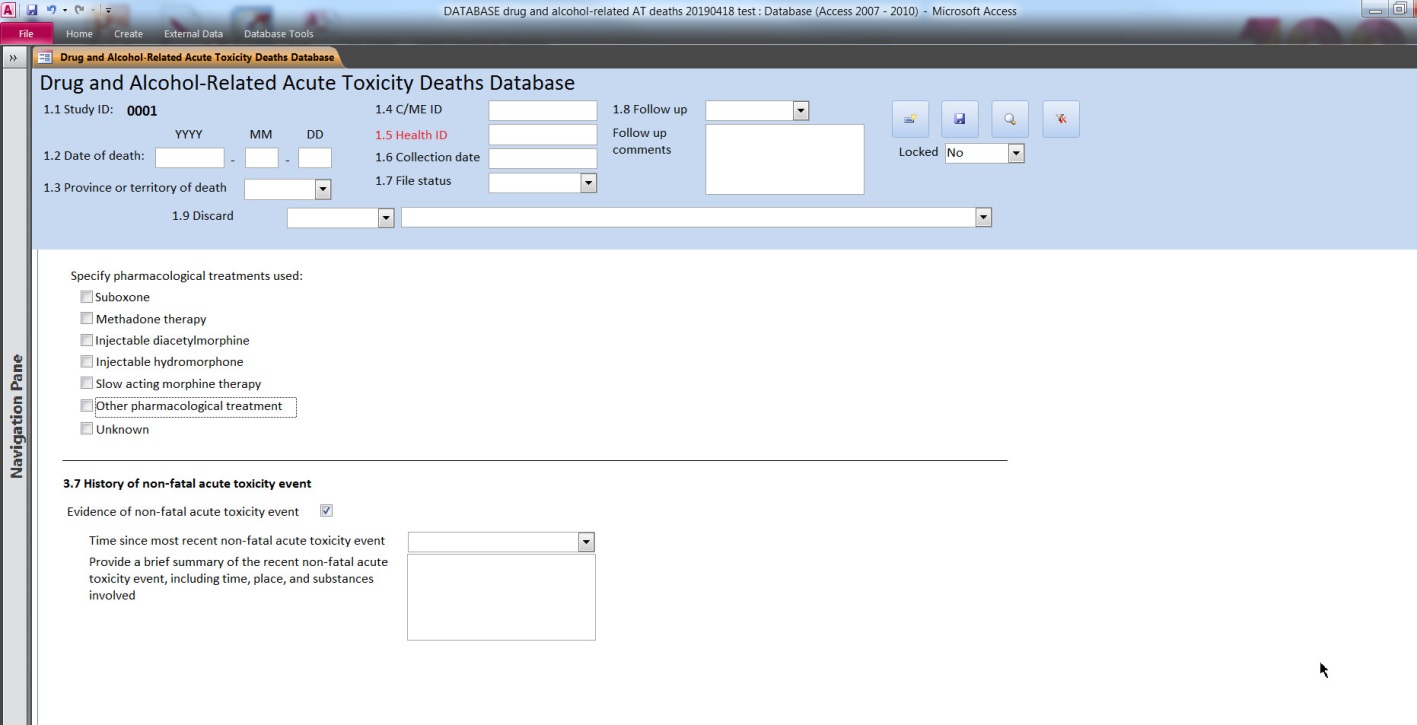
**

## Circumstances of death

Figure 6. Screenshot of the top of the circumstances of death tab of the data collection tool for our national chart review study of substance-related acute toxicity deaths. Red variables were only collected at the request of the chief coroner or medical examiner’s office and not shared with the national study team.


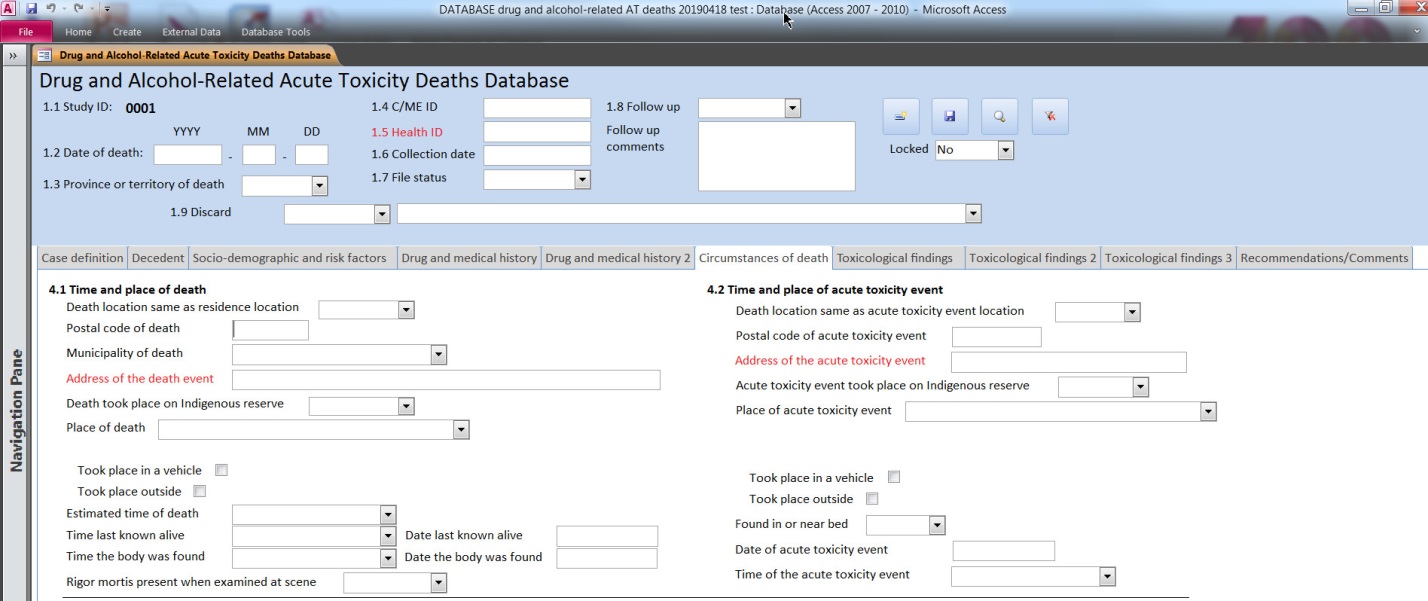


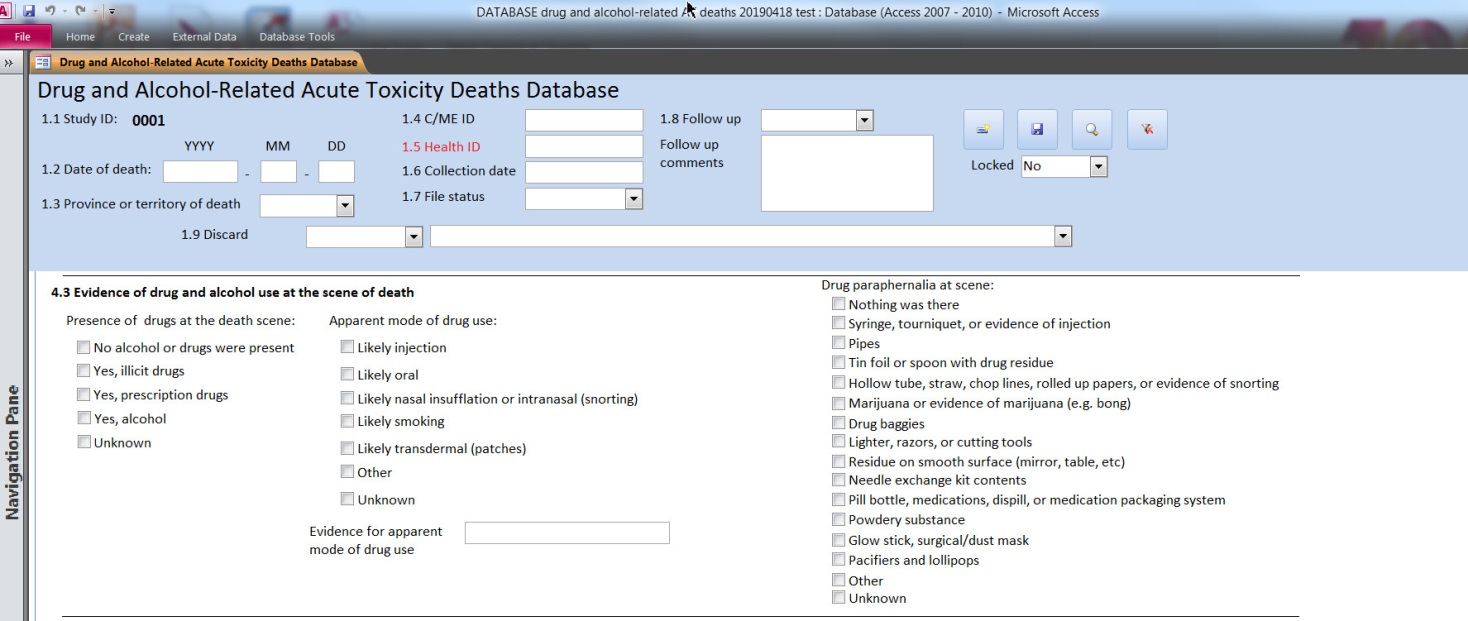


**
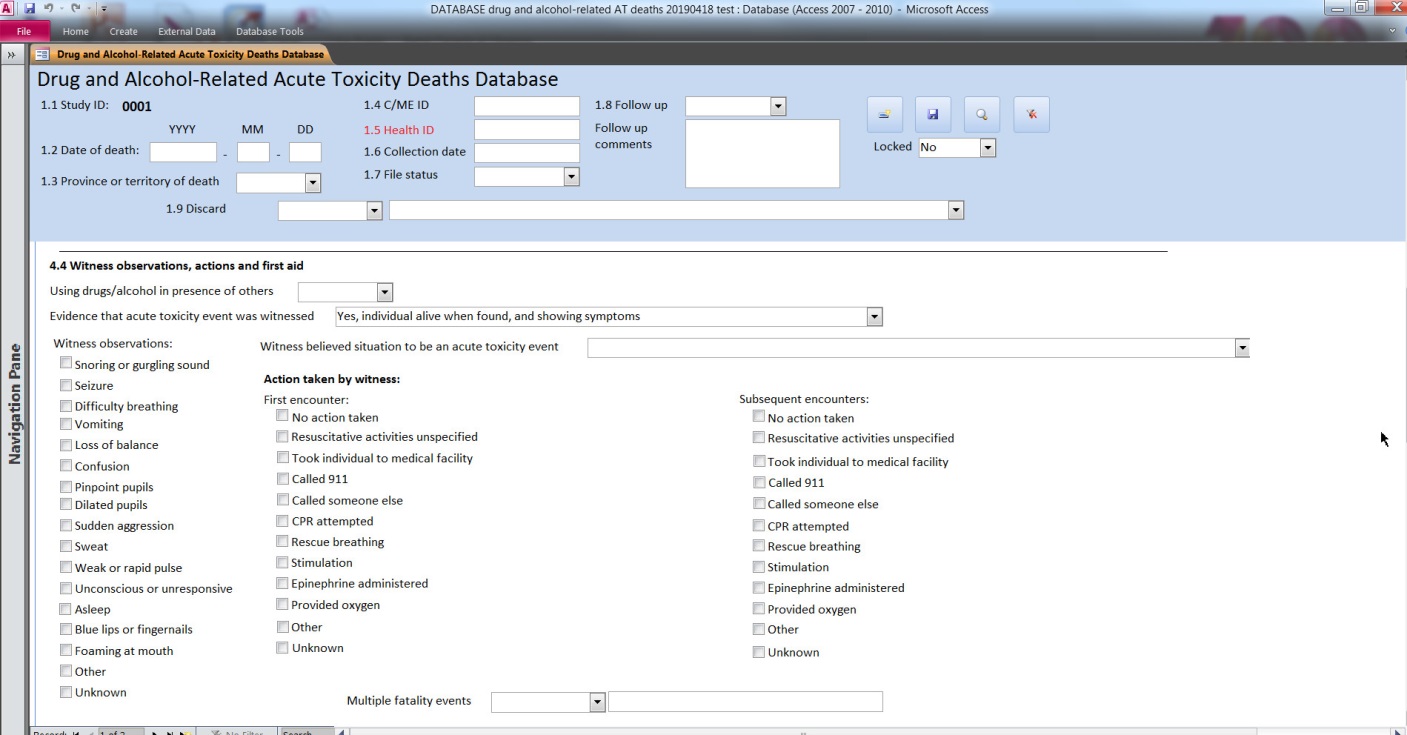
**

Figure 7. Screenshot of the bottom of the circumstances of death tab of the data collection tool for our national chart review study of substance-related acute toxicity deaths. Red variables were only collected at the request of the chief coroner or medical examiner’s office and not shared with the national study team.

**
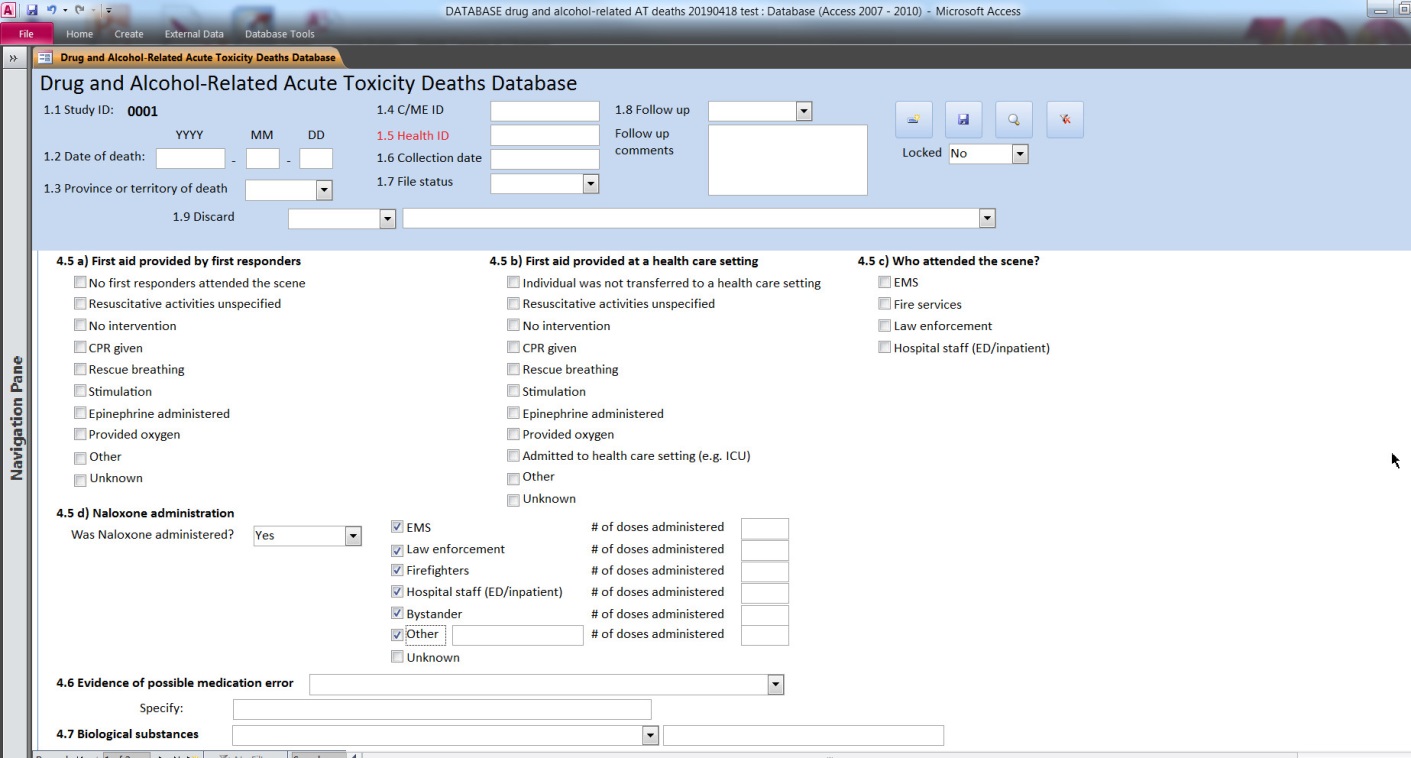
**


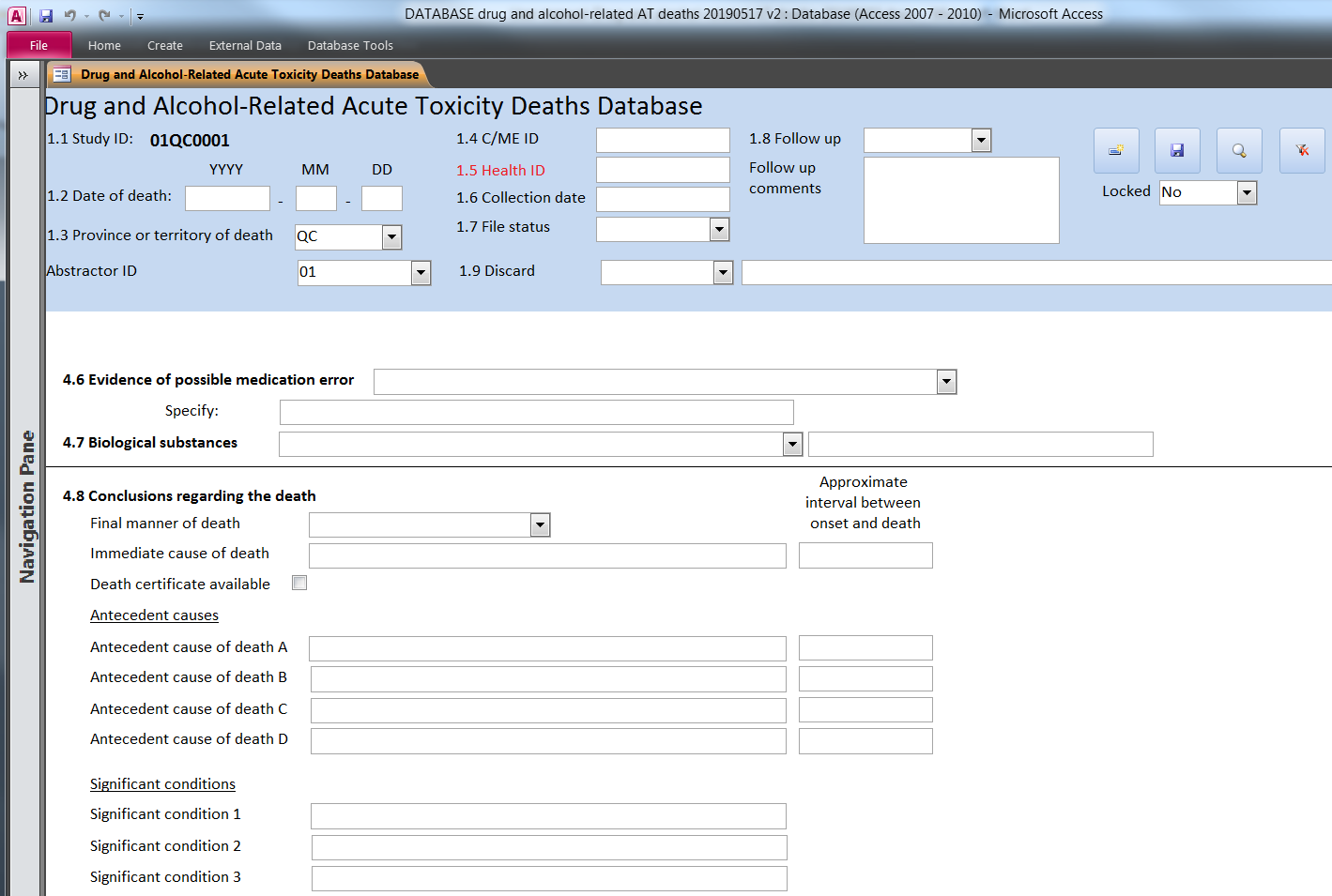


## Toxicology findings

Figure 8. Screenshot of part 1 of the toxicology findings tabs of the data collection tool for our national chart review study of substance-related acute toxicity deaths. Red variables were only collected at the request of the chief coroner or medical examiner’s office and not shared with the national study team.


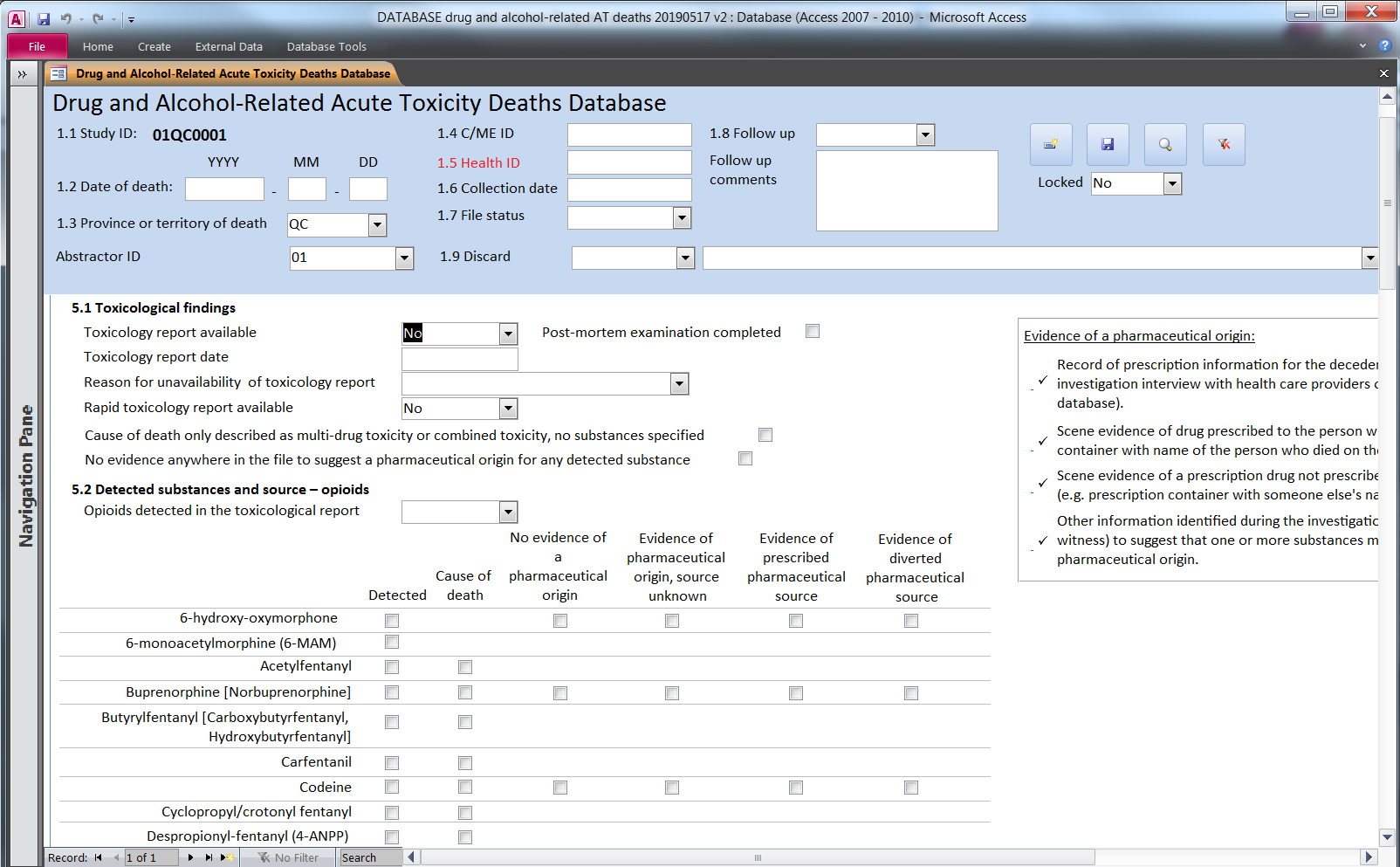


**
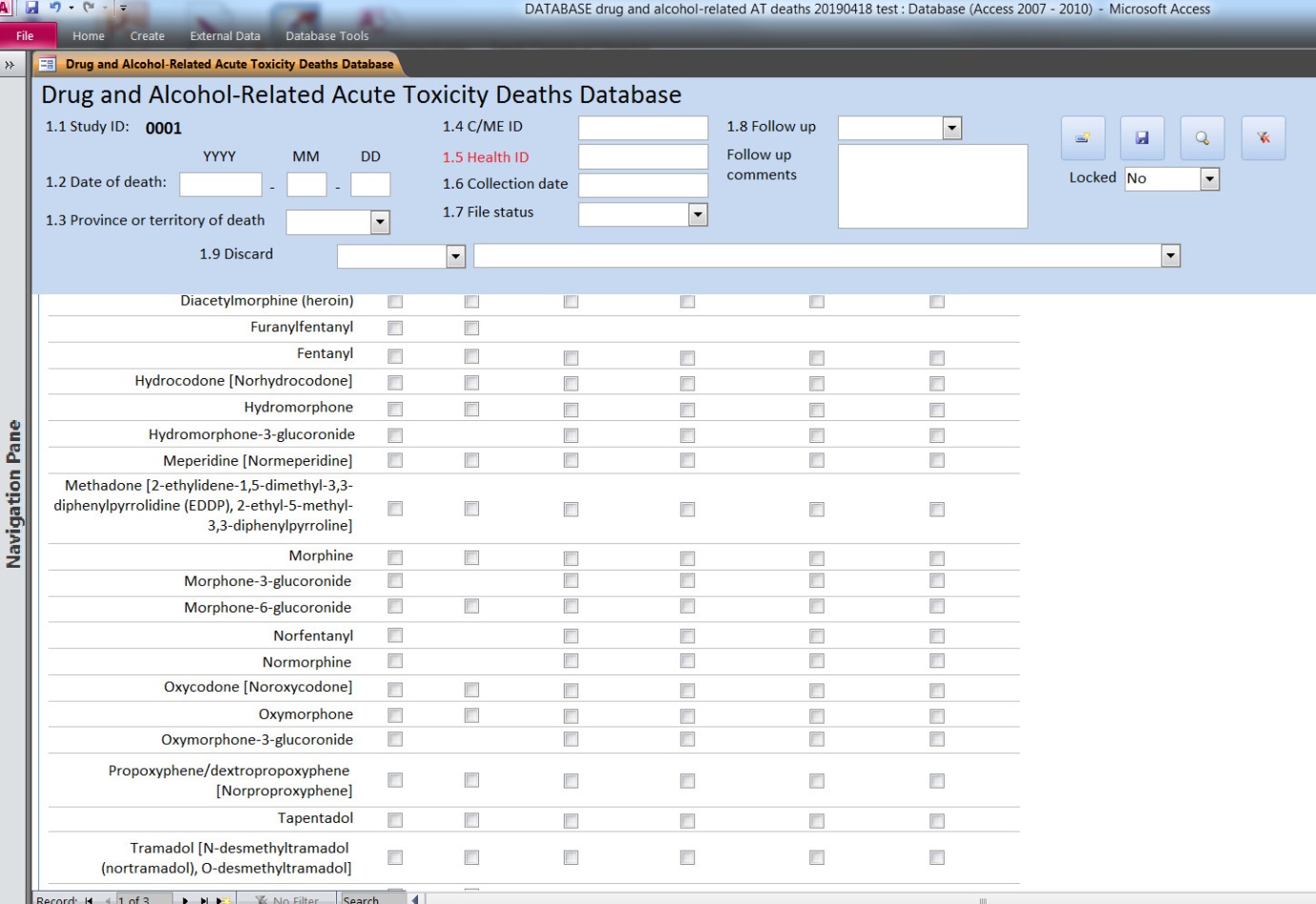
**

Figure 9. Screenshot of part 2 of the toxicology findings tabs of the data collection tool for our national chart review study of substance-related acute toxicity deaths. Red variables were only collected at the request of the chief coroner or medical examiner’s office and not shared with the national study team.

**
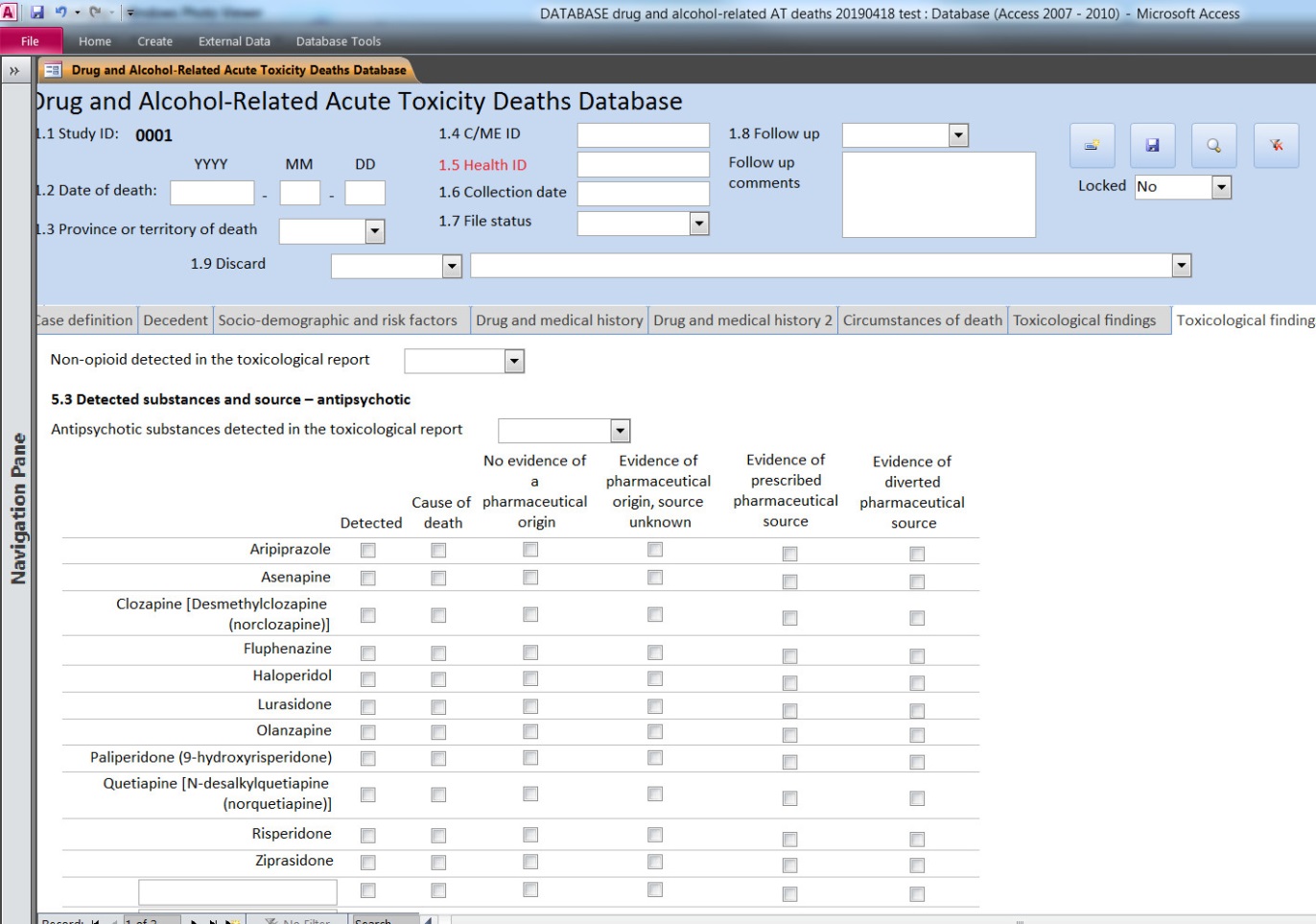
**

**
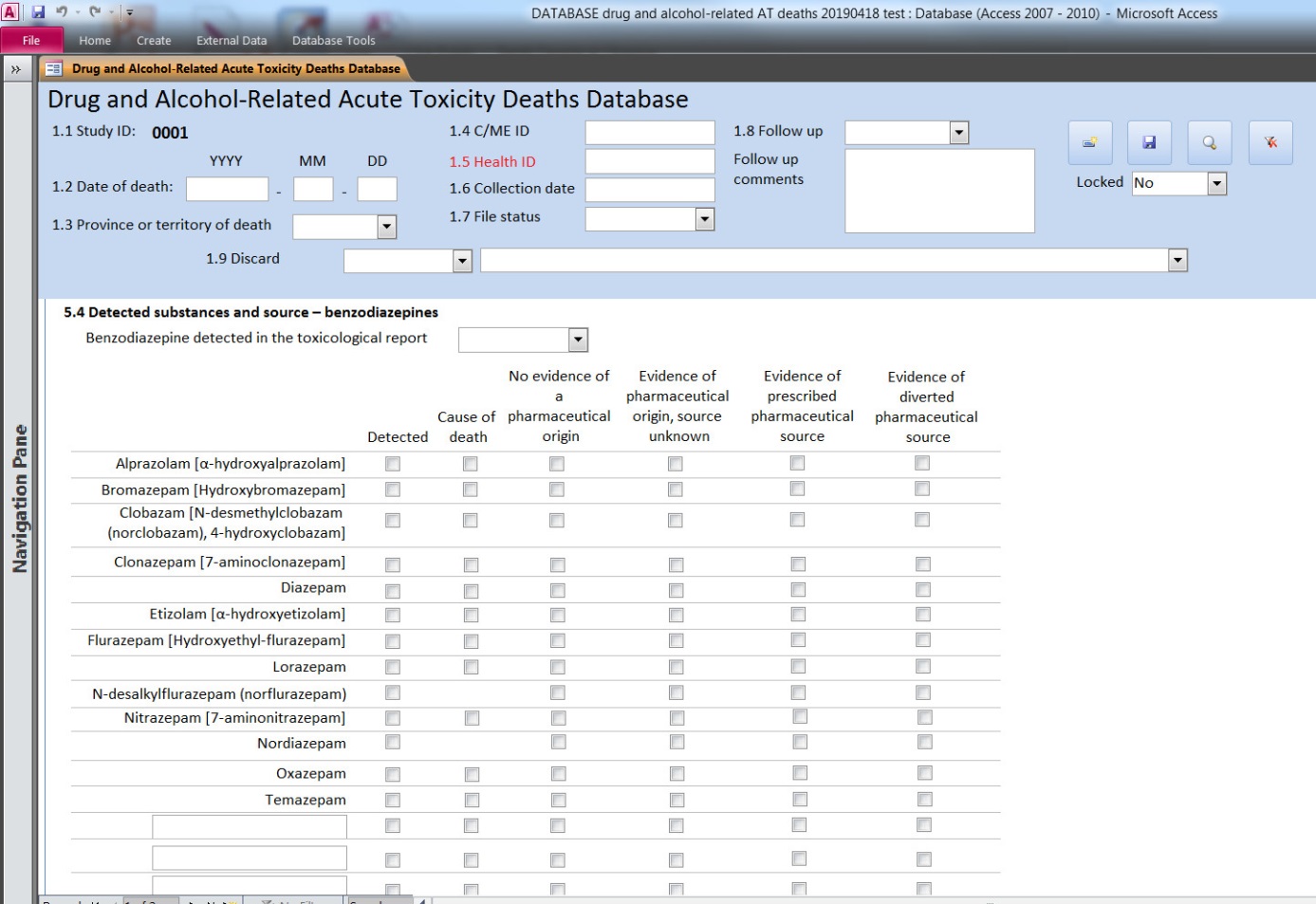
**

Figure 10. Screenshot of part 3 of the toxicology findings tabs of the data collection tool for our national chart review study of substance-related acute toxicity deaths. Red variables were only collected at the request of the chief coroner or medical examiner’s office and not shared with the national study team.

**
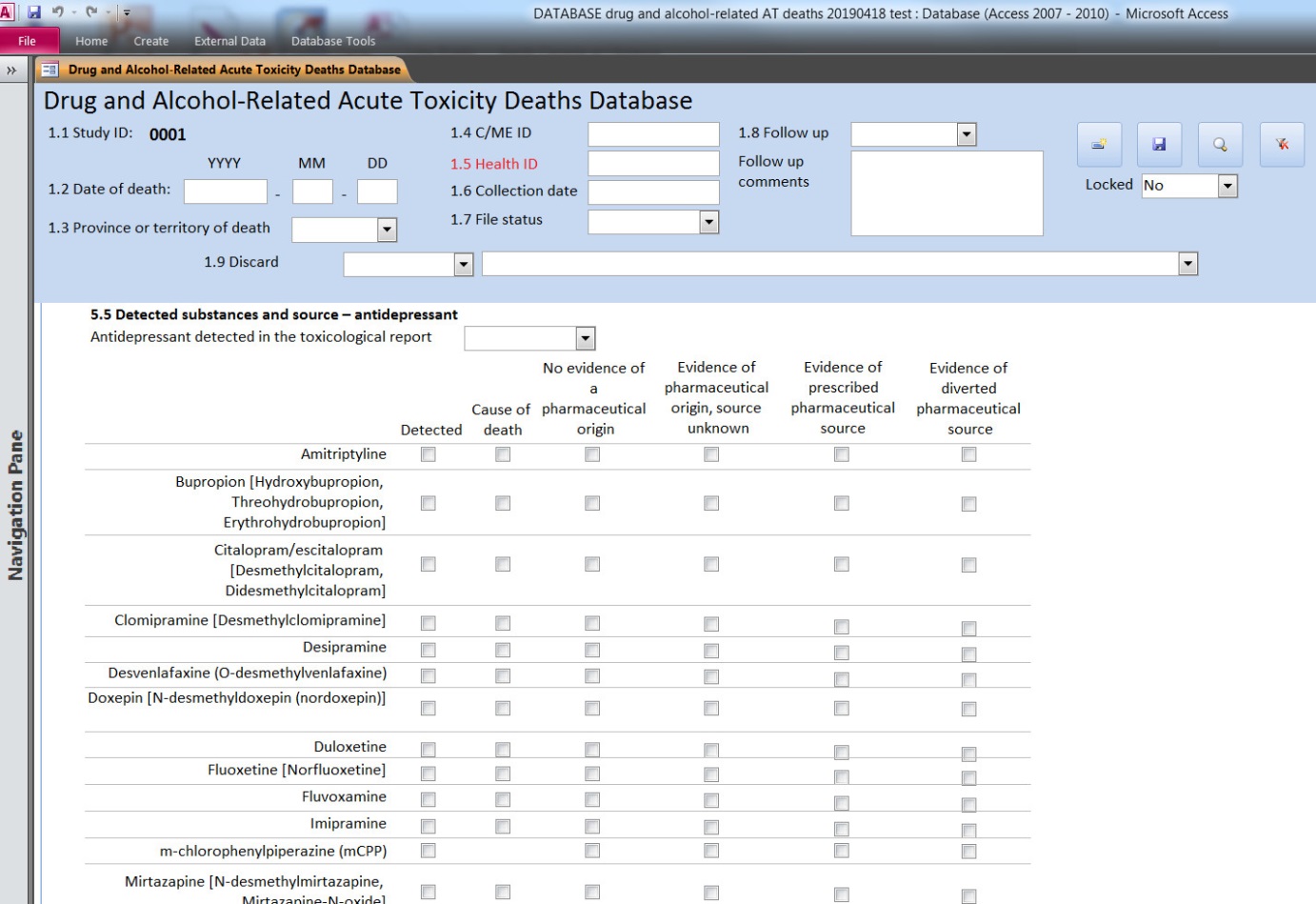
**

**
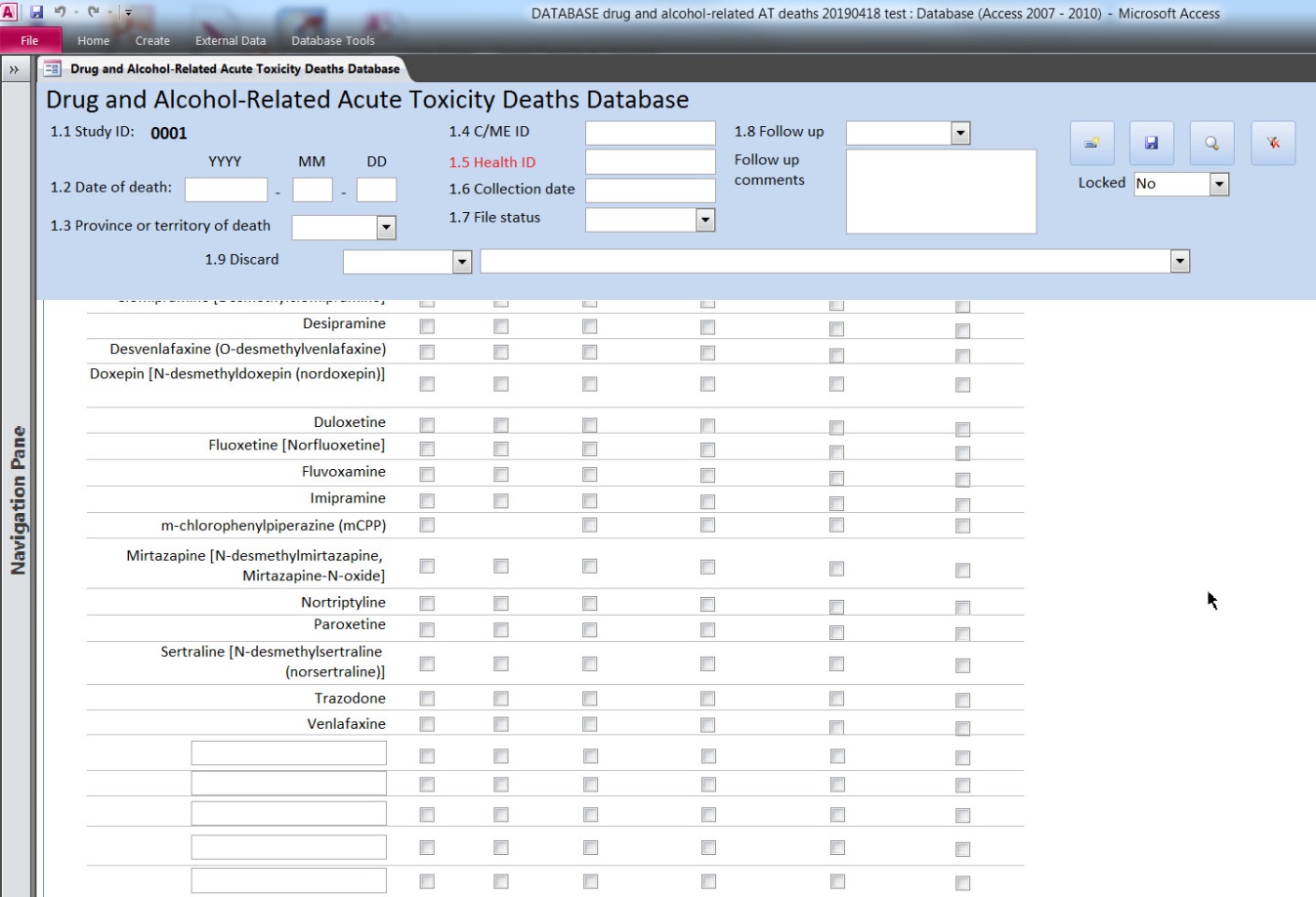
**

Figure 11. Screenshot of part 4 of the toxicology findings tabs of the data collection tool for our national chart review study of substance-related acute toxicity deaths. Red variables were only collected at the request of the chief coroner or medical examiner’s office and not shared with the national study team.

**
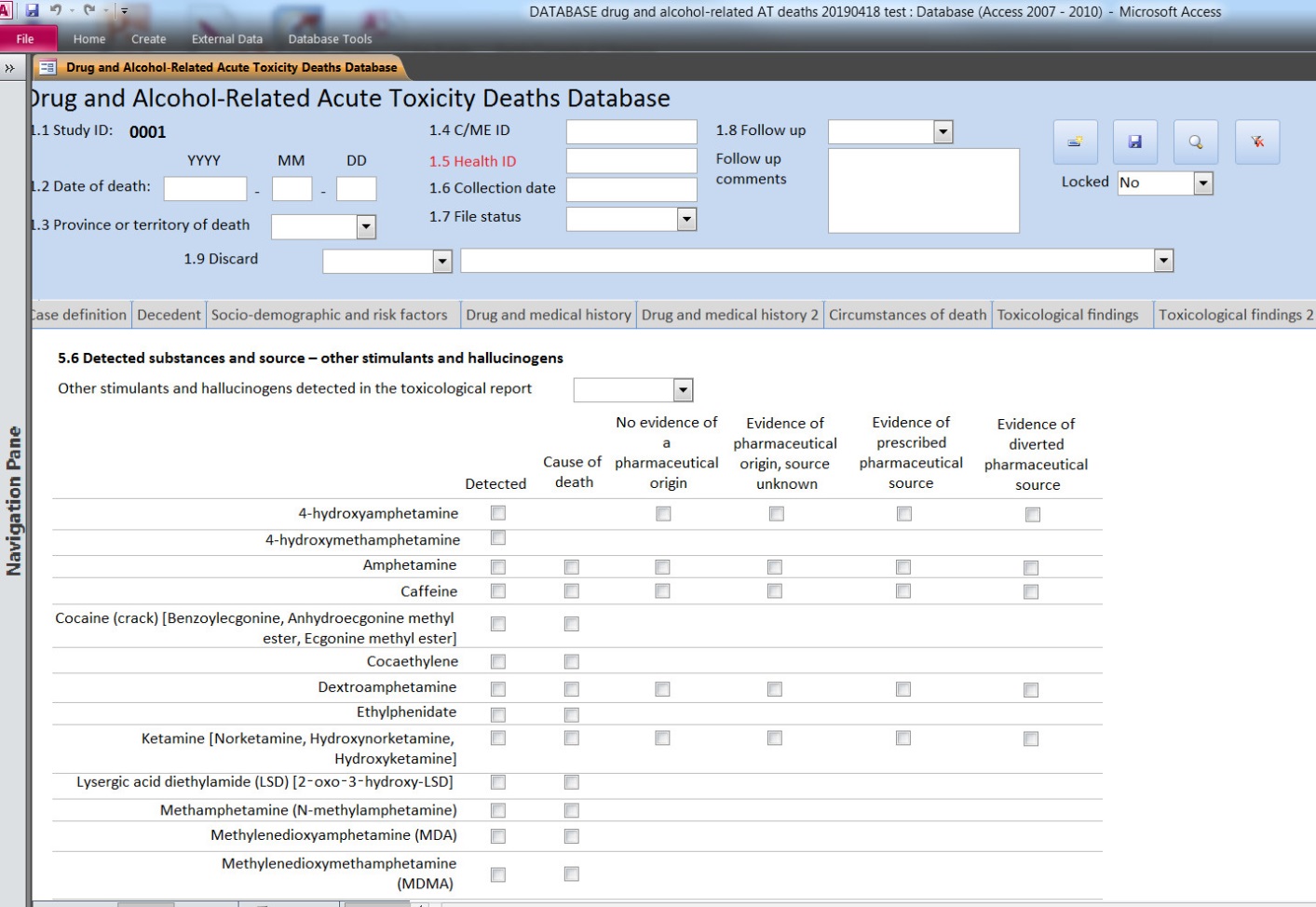
**

**
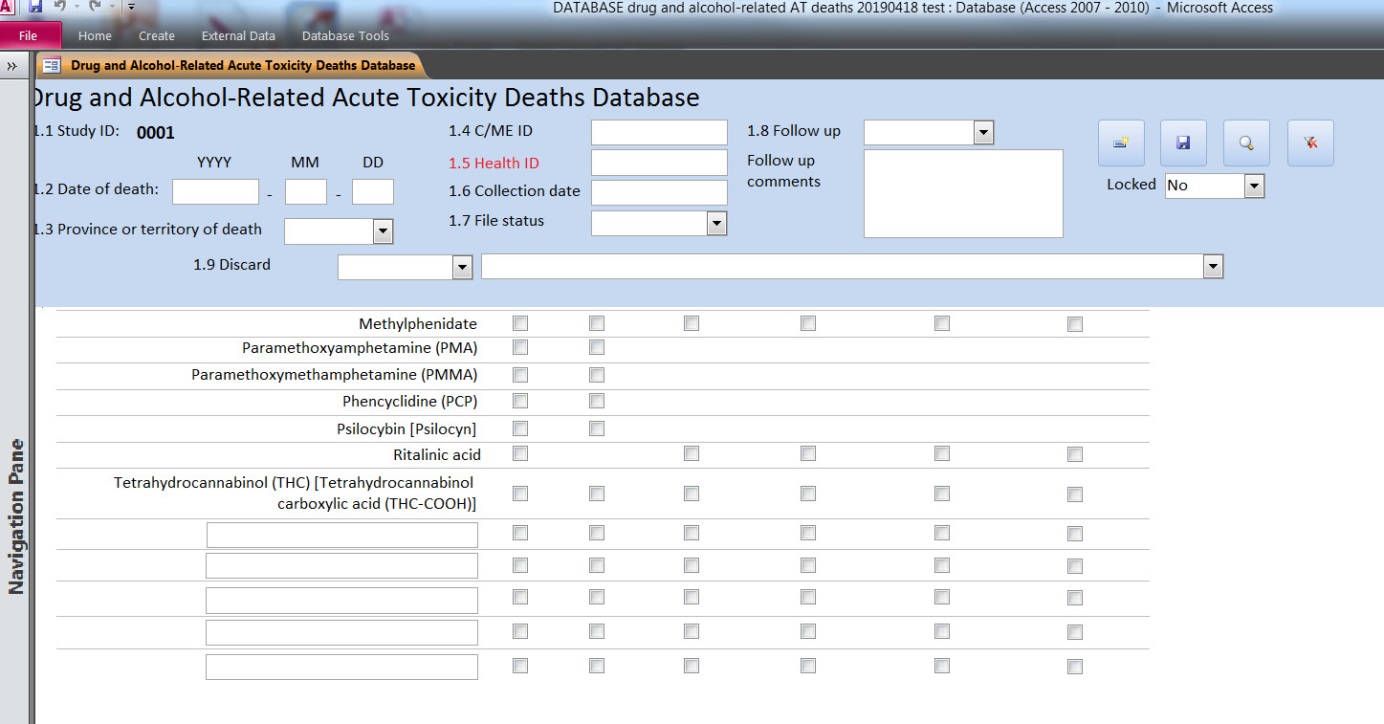
**

Figure 12. Screenshot of part 5 of the toxicology findings tabs of the data collection tool for our national chart review study of substance-related acute toxicity deaths. Red variables were only collected at the request of the chief coroner or medical examiner’s office and not shared with the national study team.

**
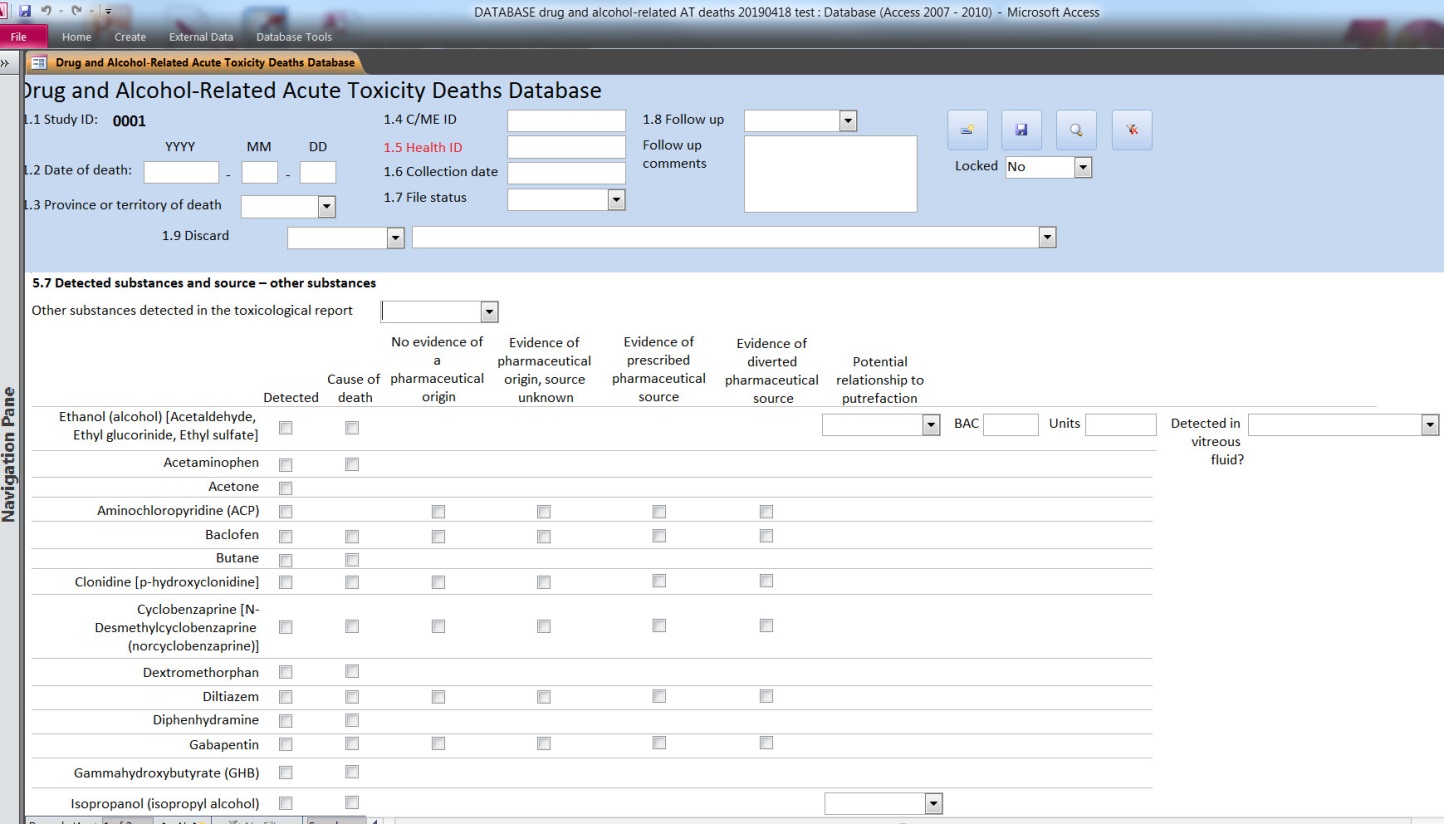
**

**
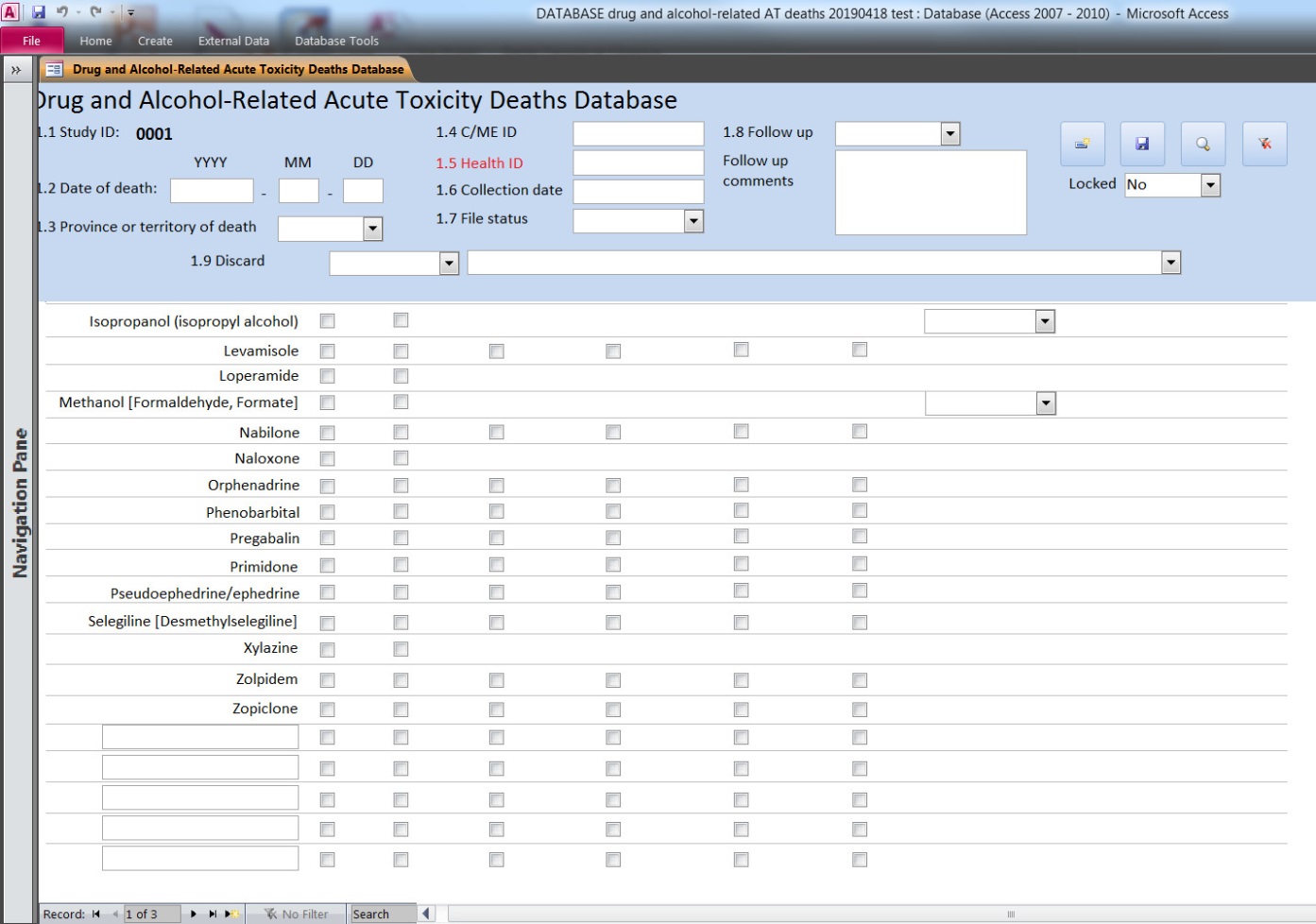
**

## Recommendations and comments

Figure 13. Screenshot of the recommendations and comments tab of the data collection tool for our national chart review study of substance-related acute toxicity deaths. Red variables were only collected at the request of the chief coroner or medical examiner’s office and not shared with the national study team.

**
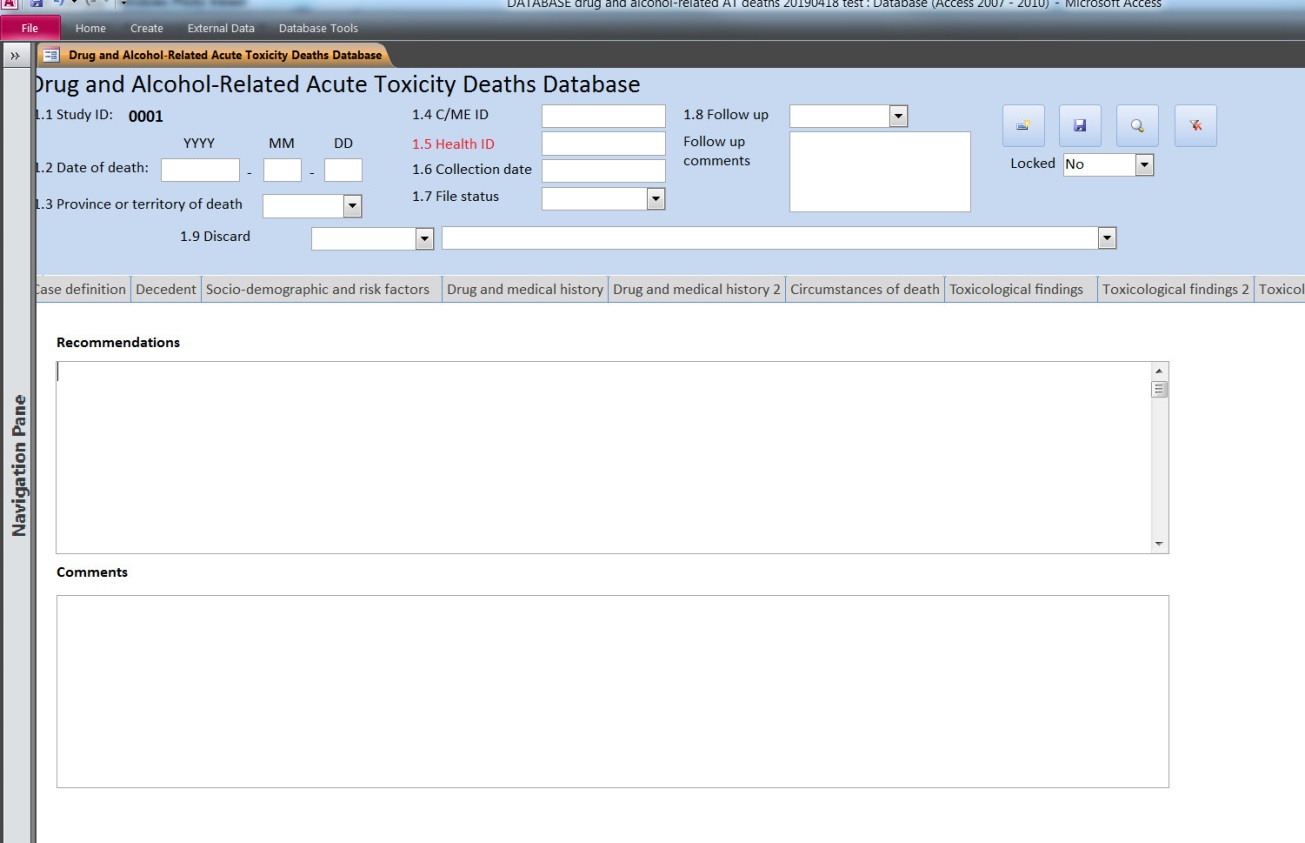
**
